# Cell death induced by the ER stressor thapsigargin involves death receptor 5, a non-autophagic function of MAP1LC3B, and distinct contributions from unfolded protein response components

**DOI:** 10.1186/s12964-019-0499-z

**Published:** 2020-01-27

**Authors:** Paula Lindner, Søren Brøgger Christensen, Poul Nissen, Jesper Vuust Møller, Nikolai Engedal

**Affiliations:** 1 Centre for Molecular Medicine Norway (NCMM), Nordic EMBL Partnership for Molecular Medicine, University of Oslo, P.O. Box 1137, Blindern, N-0318 Oslo, Norway; 2 Danish Research Institute of Translational Neuroscience (DANDRITE), Nordic EMBL Partnership for Molecular Medicine, Department of Molecular Biology and Genetics, Aarhus University, Aarhus, Denmark; 3 Department of Drug Design and Pharmacology, University of Copenhagen, Copenhagen, Denmark; 4 Department of Biomedicine, Aarhus University, Aarhus, Denmark

**Keywords:** Thapsigargin, SERCA, Unfolded protein response, DR5, Caspase-8, PERK, ATF4, CHOP, IRE1, XBP1s, JNK, LC3B, Cell death, Apoptosis, Autophagy

## Abstract

**Background:**

Cell death triggered by unmitigated endoplasmic reticulum (ER) stress plays an important role in physiology and disease, but the death-inducing signaling mechanisms are incompletely understood. To gain more insight into these mechanisms, the ER stressor thapsigargin (Tg) is an instrumental experimental tool. Additionally, Tg forms the basis for analog prodrugs designed for cell killing in targeted cancer therapy. Tg induces apoptosis via the unfolded protein response (UPR), but how apoptosis is initiated, and how individual effects of the various UPR components are integrated, is unclear. Furthermore, the role of autophagy and autophagy-related (ATG) proteins remains elusive.

**Methods:**

To systematically address these key questions, we analyzed the effects of Tg and therapeutically relevant Tg analogs in two human cancer cell lines of different origin (LNCaP prostate- and HCT116 colon cancer cells), using RNAi and inhibitory drugs to target death receptors, UPR components and ATG proteins, in combination with measurements of cell death by fluorescence imaging and propidium iodide staining, as well as real-time RT-PCR and western blotting to monitor caspase activity, expression of ATG proteins, UPR components, and downstream ER stress signaling.

**Results:**

In both cell lines, Tg-induced cell death depended on death receptor 5 and caspase-8. Optimal cytotoxicity involved a non-autophagic function of MAP1LC3B upstream of procaspase-8 cleavage. PERK, ATF4 and CHOP were required for Tg-induced cell death, but surprisingly acted in parallel rather than as a linear pathway; ATF4 and CHOP were independently required for Tg-mediated upregulation of death receptor 5 and MAP1LC3B proteins, whereas PERK acted via other pathways. Interestingly, IRE1 contributed to Tg-induced cell death in a cell type-specific manner. This was linked to an XBP1-dependent activation of c-Jun N-terminal kinase, which was pro-apoptotic in LNCaP but not HCT116 cells. Molecular requirements for cell death induction by therapy-relevant Tg analogs were identical to those observed with Tg.

**Conclusions:**

Together, our results provide a new, integrated understanding of UPR signaling mechanisms and downstream mediators that induce cell death upon Tg-triggered, unmitigated ER stress.

Video Abstract

**Graphical abstract:**

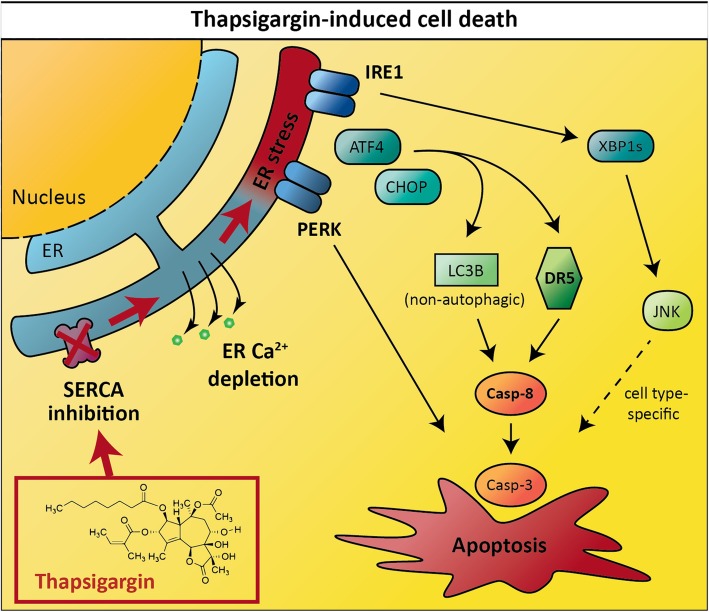

## Background

Insufficient capacity of the endoplasmic reticulum (ER) to fold newly synthesized proteins leads to accumulation of unfolded proteins in the ER; a situation referred to as “ER stress”. In response to ER stress, the cell initiates the “unfolded protein response” (UPR), which by different means aims to restore the balance between ER folding capacity and unfolded protein load. If the cell fails to restore this balance, the resulting chronic ER stress and persistent UPR signaling leads to cell death [1]. Cell death induced by unresolved ER stress is implicated in a growing list of pathophysiological conditions, including neurological and cardiovascular diseases, ophthalmology disorders, viral infections, and diabetes [2–4]. In many of these cases, the pathophysiology is linked to decreased levels of ER Ca^2+^ [3], which leads to ER stress and UPR due to the dependency of the ER protein folding machinery on ER lumenal Ca^2+^ [5, 6]. If we can understand the molecular mechanisms that initiate cell death under such conditions, it can lay the ground for the development of novel treatment modalities for various diseases.

Experimentally, ER Ca^2+^ depletion and a resulting UPR can be achieved by use of the specific sarco/endoplasmic reticulum Ca^2+^-ATPase (SERCA) inhibitor thapsigargin (Tg; for chemical structure see Additional file 1: Figure S1A) - a classical tool to study ER stress and UPR biology. Tg produces sustained and unmitigated ER stress in mammalian cells, leading to UPR-induced apoptosis [7–13]. Besides its use as an experimental tool, Tg is the mother compound of prodrugs designed for use in targeted cancer therapy [14–16]. In the current study, we set out to explore the signaling mechanisms of cell death induction by Tg and Tg analogs derived from Tg prodrugs (Additional file 1: Figure S1A) in two different cancer cell lines (LNCaP and HCT116), with the overarching aims to: (i) enhance our understanding of ER stress-induced signaling to cell death with a particular relevance to diseases associated with UPR-induced apoptosis and decreased levels of ER Ca^2+^, and (ii) better understand the mechanisms of action of therapeutically relevant Tg analogs. We focused in particular on defining upstream cell death-initiating events and how the various components of the UPR integrate on them – an important area of investigation wherein, as described below, many key questions are unresolved.

Multiple studies indicate that unmitigated ER stress initiates cell death via the extrinsic pathway. Tg and other ER stressors frequently upregulate the mRNA and protein levels of death receptor 5 (DR5; TRAIL-R2) [9, 13, 17–22] and initiate DR5- [9, 13, 18–21] and caspase-8- [9, 18, 21–25] dependent cell death in various cell types. However, death receptors other than DR5 have also been implicated in ER stress-induced apoptosis, including death receptor 1 (DR1; TNFR1) [26], death receptor 2 (DR2; Fas) [27], and death receptor 4 (DR4; TRAIL-R1) [19, 20]. The relative importance of the various death receptors in ER stress-induced cell death is unknown, since, to the best of our knowledge, no single study has directly compared their involvements. In the current study, we found Tg-induced upregulation of DR5 to be strongly required for activation of caspase-8 and cell death in both LNCaP and HCT116 cells, while other death receptors played a minor or no role.

Autophagy and autophagy-related (ATG) proteins are frequently implicated in cell death regulation [28], but their role in ER stress-induced cell death is unclear. ER stress potently upregulates the expression of microtubule-associated proteins 1A/1B light chain 3B (LC3B), as well as other ATGs [29–33]. This correlates with enhanced autophagic flux in cells treated with the ER stressor tunicamycin (Tm) [30], whereas Tg blocks general autophagy due to ER Ca^2+^ depletion [11, 33]. Variable and inconsistent results have been reported with respect to the effect of genetic depletion of key ATGs (Beclin1 or ATG5) on Tg- and Tm-induced cell death [25, 34–37]. Thus, additional research is needed to define the role of autophagy and ATGs in ER stress-induced cell death. In both LNCaP and HCT116 cells, we found LC3B, but not other central ATGs (ATG5, FIP200, GABARAPs), to be required for optimal Tg-induced cell death. Moreover, LC3B acted upstream of caspase-8 activation.

Having defined ER stress-induced DR5 and LC3B upregulation as important cell death-initiating signaling events, we aimed to delineate the individual contributions from the different UPR components to DR5 and LC3B expression, as well as to caspase activation and cell death. The UPR is classically divided into the PERK, IRE1, and ATF6 arms [1, 38]. Upon accumulation of unfolded proteins in the ER, the protein kinase PERK is activated via dimerization and trans-autophosphorylation. Active PERK subsequently phosphorylates downstream targets, including eukaryotic initiation factor 2α. The latter event leads to a transient block in general protein synthesis, while favoring translation of selected transcripts, including ATF4. ATF4 is a transcription factor that controls the expression of numerous genes, including the transcription factor CHOP. Upon ER stress, IRE1 dimerizes/oligomerizes, trans-autophosphorylates, and acquires RNase activity. The latter results in alternative splicing of XBP1, producing the transcription factor XBP1s. Additionally, the IRE1 RNase activity triggers the cleavage and degradation of a broad range of transcripts via a mechanism termed regulated IRE1-dependent decay (RIDD). IRE1 can also interact with TRAF2 and ASK1, leading to activation of c-Jun N-terminal kinase (JNK) [39, 40]. Finally, upon ER stress, ATF6 translocates to the Golgi apparatus, where proteolytic cleavage releases the ATF6 transcription factor fragment.

CHOP has been demonstrated to play an important role in ER stress-induced DR5 upregulation and cell death [9, 13, 18], but the precise roles of PERK and ATF4 are yet to be experimentally determined. It is important to point out that, even though often assumed, the PERK-ATF4-CHOP axis is not necessarily a strictly linear pathway. In fact, under some circumstances, ER stress-induced elevation of ATF4 protein levels can occur independently of PERK [30] and ER stress-mediated induction of CHOP can occur independently of ATF4 [41]. Moreover, PERK can exert cellular effects via other mechanisms than through ATF4 [30, 38, 41–44], and ATF4 has many target genes other than CHOP [41, 45]. Consequently, careful experimental study is required to determine the individual roles of PERK, ATF4, and CHOP in ER stress-induced regulation of cell death and DR5 expression. The same applies to the regulation of LC3B expression, where separate studies have implicated various components of the PERK branch [30, 32, 36, 46], but where the delineation of the individual roles played by PERK, ATF4 and CHOP is incomplete.

The role of IRE1 in ER stress-induced cell death is complex and yet to be fully understood. A pro-survival function of IRE1-XBP1 signaling has been suggested, since forced enhancement of IRE1-mediated XBP1 splicing can protect cells from ER stress-induced apoptosis [47]. Moreover, XBP1 depletion can sensitize cells that are exposed to low-level cytotoxic ER stress [48]. However, the precise role played by the endogenously activated IRE1-XBP1 pathway during unopposed, cytotoxic ER stress is yet to be defined. Several studies indicate that IRE1 contributes to ER stress-induced cell death via its RIDD activity [49–51]. However, another study proposed that specific IRE1 RIDD activity towards DR5 mRNA opposes ER stress-induced cell death [9]. Finally, a number of studies suggest that IRE1 can contribute to ER stress-induced cell death via activation of JNK [39, 52–54]. Contrasting results have been reported with regard to the overall, net effect of IRE1 on ER stress-induced cell death [9, 18, 49], and opposing results obtained with the same ER stressor (Tg) in different cell lines [9, 18, 49] suggest a cell type-dependent role of IRE1. However, molecular explanations for such cell type-specific differences are lacking. The role of ATF6 in ER stress-induced cell death has been much less studied than the two other UPR branches. However, a pro-survival role for ATF6 has been observed in response to chronic, mild ER stress [55], and knockdown of ATF6 was reported to sensitize melanoma cells to apoptosis induction by Tm or Tg [56]. Apart from the influence of IRE1 on DR5 described above [9], the roles of IRE1, XBP1, and ATF6 in regulating DR5 and LC3B expression during death-inducing ER stress stimuli have not been examined previously.

In this study, we undertook a systematic RNAi-based approach to delineate the roles of the UPR components in Tg-induced regulation of DR5 and LC3B expression, and cell death. We found ATF4 and CHOP to be individually required for the upregulation of both DR5 and LC3B, as well as for cell death, whereas PERK was required for cell death but not for upregulation of DR5 and LC3B protein. ATF6 knockdown neither reduced cell death nor DR5 or LC3B expression. IRE1 and XBP1 were partially required for Tg-induced cell death in LNCaP but not HCT116 cells, and partially required for upregulation of DR5, but not LC3B. Notably, we found that IRE1 activated JNK in an XBP1-dependent manner, and JNK was required for Tg-induced cell death in LNCaP cells, without affecting DR5 or LC3B expression. The inability of IRE1-XBP1 to influence Tg-induced cell death in HCT116 cells was associated with Tg-induced cell death being independent of JNK in this cell line.

Lastly, we examined the mechanisms of cell death induction by active components of therapeutically relevant Tg prodrugs. Inactive, cell impermeable Tg prodrugs contain a 12-aminododecanoyl linker with specific peptide sequences that are cleaved by tumor-produced proteolytic enzymes, thereby generating active, cell permeable Tg analogs only in the vicinity of the tumor [14–16]. Tg prodrugs have shown impressive tumor-eradicating effects in preclinical animal models [14, 15, 57, 58]. Moreover, one prodrug has reached the stage of clinical phase II trials, where it recently was demonstrated to promote prolonged disease stabilization in patients with advanced hepatocellular carcinoma [59]. Prodrug-derived Tg analogs induce apoptotic cell death correlated with sustained UPR induction in cancer cells [10], but other than that little is known about how the effects of the Tg analogs compare to those of Tg. Strikingly, our focussed RNAi-based screening approach revealed virtually identical molecular requirements for cell death induced by therapeutically relevant Tg analogs as those we had identified for Tg, in both LNCaP and HCT116 cells.

## Materials and methods

### Chemicals

Thapsigargin (Tg) was purchased from Sigma (T9033). The Tg analogs 8-*O*-debutanoyl-8-*O*-*N*-L-Leucinoyl-12-aminododecanoylthapsigargin (Leu-8ADT) and 8-*O*-debutanoyl-8-*O*-*N*-L-β-aspartoyl-12-aminododecanoylthapsigargin (βAsp-8ADT) were synthesized as previously described [60]. The PERK inhibitor GSK2606414 [61] was a kind gift from Dr. Jeffrey M. Axten (GlaxoSmithKline). The JNK inhibitor JNK-IN-8 was from Sigma (SML1246). The chemical structures of Tg, Leu-8ADT, βAsp-8ADT, GSK2606414, and JNK-IN-8 are depicted in Additional file 1: Figure S1.

### Cell culture and siRNA transfections

LNCaP prostate cancer cells (ATCC, CRL-1740) and HCT116 colorectal carcinoma cells (ATCC, CCL-247) were cultured in RPMI 1640 (Thermo Fisher Scientific, 21875091) or DMEM/F12 (Thermo Fischer Scientific, 11320074) medium, respectively, containing 10% FBS (Sigma, F7524 batch BCBT0730) at 37 °C in a humidified incubator containing 5% CO_2_. RNAi -mediated knockdown was performed by reverse transfection, using RNAiMax (Invitrogen 13778) and 5 nM siRNA, according to the manufacturer's protocol. The following Silencer Select siRNAs (Ambion) were used: siCtrl (non-targeting negative Control, 4390843), siATF4–1 (s1704), siATF4–2 (s1702), siATF6–1 (s223544), siATF6-2 (s223545), siCaspase8–1 (s2425), siCaspase8–2 (s2427), siCHOP-1 (s3996), siCHOP-2 (s3997), siDR4 (s16764), siDR5–1 (s225037), siDR5–2 (s16756), siFADD (s228426), siFas (s223933), siGABARAP (s22361), siGABARAPL1 (s24332), siGABARAPL2 (s22385), siFIP200 (s18995), siIRE1–1 (s200432), siIRE1–2 (s200430), siLC3B-1 (s37748), siLC3B-2 (s224886), siPERK-1 (s18101), siPERK-2 (s18103), siTRADD (s16607), siXBP1–1 (s14913), siXBP1–2 (s14914), and the following ON-TARGET plus siRNAs from Darmacon: siCtrl (non-targeting negative Control, D-001810-01–20) and siATG5 (J-004374-07).

### Cell death measurements

Cell death was assessed by live-cell fluorescence imaging of propidium iodide-stained cells as previously described [10, 62]. Briefly, cells were seeded in triplicates in 96-well plates and grown to ~ 60–80% confluency. Treatments were added along with 2.5 μg/ml (final concentration) propidium iodide to stain for dead cells, and the IncuCyte ZOOM imaging system (Essen Bioscience) was used for live-cell phase-contrast and fluorescence imaging, acquiring 3 images from each well every 3 h. The integrated software was used to calculate ratios of red fluorescence- to total cell confluency, as previously described [10, 62]. The data was normalized to the average ratio from each experiment. Relative cell death compared to Tg-treated control cells was quantified at 48 h (LNCaP) or 39 h (HCT116). These time points represent the boundary between the linear and asymptotic regions in the typical sigmoid curves that are obtained upon plotting cell death versus time in Tg-treated cells [10, 62], and when more than 50% of the cells were dead in the Tg-treated siCtrl-transfected condition [10, 62] (and data not shown).

### Immunoblotting

Cells were harvested after 30 h of treatment with Tg, unless otherwise stated. Preparation of whole-cell lysates, SDS-PAGE, and immunoblot analysis were performed as described previously [33]. The following antibodies were used: alpha-tubulin (Abcam, ab7291), ATG5 (Cell Signaling Technology 2630), ATF4 (Cell Signaling Technology 11815), Bip/Grp78 (Cell Signaling Technology 3177), Caspase-3 (8G10) (Cell Signaling Technology 9665), Caspase-8 (1C12) (Cell Signaling Technology 9746), CHOP (Cell Signaling Technology 2895), Cleaved PARP (Asp214) (D64E10) (Cell Signaling Technology 5625), DR5 (Cell Signaling Technology 8074), FADD (G-4) (Santa Cruz sc-271748), FIP200 (Proteintech 17250–1-AP, GABARAP (Proteintech PM037), GABARAPL1 (Abcam ab86497), GABARAPL2 (Proteintech PM038), p-JNK (Cell Signaling Technology 9251), JNK (Cell Signaling Technology 9252), LC3B (Cell Signaling Technology 2775), PERK (Cell Signaling Technology 5683), XBP1s (BioLegend 647502).

### Real-time RT-PCR

Total RNA was isolated from cell pellets using the ReliaPrep RNA Miniprep Systems (Promega) according to manufacturer's instructions. Reverse transcription was performed using SuperScript VILO Master Mix (Applied Biosystems 11,755,250) and subsequently mRNA levels were determined by real-time PCR using TaqMan gene expression assays (Appl. Biosys. 11755250) with TaqMan fast advanced PCR master mix (Appl. Biosys. 4444558). PCR amplification was done in triplicate measurements per sample with the ABI 7900HT FAST sequence detection system (Appl. Biosys.). The cycling conditions were 50 °C for 2 min, 95 °C for 10 min, 40 cycles at 95 °C for 15 s and 60 °C for 1 min. Relative mRNA levels were normalization to the geometric mean CT value of GAPDH and TBP, and thereafter calculated by the comparative CT method compared to the DMSO treated control cells (set as 1). The following TaqMan probes (Appl. Biosys.) were used: ATF4 Hs00909569_g1, ATF6 Hs0023586_m1, DDIT3 (CHOP) Hs00358796_g1, EIF2AK3 (PERK) Hs00984003_m1, ERN1 (IRE1) Hs00176385_m1, FAS Hs00236330_m1, GAPDH Hs99999905_m1, MAP1LC3B Hs00797944_s1, TBP Hs99999910_m1, TNFRSF10A (DR4) Hs00269492_m1, TNFRSF10B (DR5) Hs00366278_m1, TRADD Hs00601065_g1, XBP1 Hs02856596_m1, XBP1s Hs0329085_g1.

### Statistics and calculation of RNAi-mediated effects on cell death and protein expression

Data are reported as mean of at least 3 independent experiments with SEM as error bars, unless otherwise indicated. To determine significance levels, repeated measures one-way analysis of variance (ANOVA) with Dunnett’s multiple comparison test was performed using GraphPad Prism 8. Significance was assumed if *P* < 0.05. The effect of a specific siRNA, here denoted as “siX”, on Tg-induced cell death or upregulation of protein expression, as compared to the basal levels observed in the siCtrl+DMSO condition, was calculated as follows: The difference between the average values obtained in the siCtrl+Tg condition versus the siX+Tg condition was divided by the difference between the average values obtained in the siCtrl+Tg condition versus the siCtrl+DMSO condition, and next multiplied by 100 to present the percentage effect.

## Results

### DR5 and caspase-8 are main initiators of Tg-induced cell death in LNCaP and HCT116 cells

**Fig. 1:**
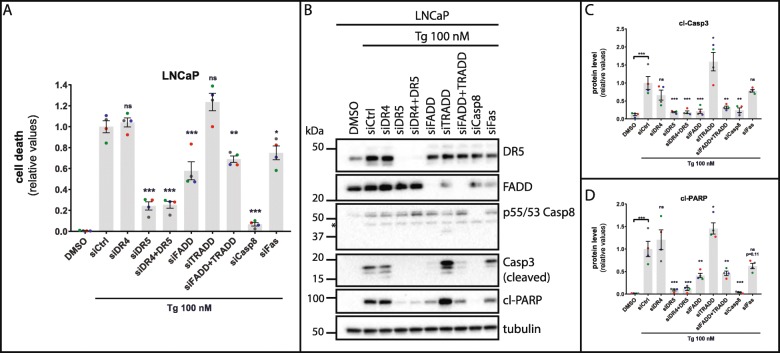
Tg-induced cell death depends on DR5 and caspase-8, and partially requires FADD and Fas, but not DR4 or TRADD in LNCaP cells. **a** LNCaP cells were transfected for 2 d with the indicated siRNAs (siCtrl = non-targeting control siRNA, siCasp8 = siCaspase-8). Subsequently, cells were treated with 100 nM Thapsigargin (Tg) or 0.01% DMSO vehicle control (“DMSO”, also transfected with siCtrl) in the additional presence of 2.5 μg/ml propidium iodide to stain dead cells. Cell death was monitored and quantified with the IncuCyte ZOOM as described in Materials and Methods, and displayed as relative values normalized to those obtained in the siCtrl+Tg condition after 48 h of treatment (mean value set to 1). **b** LNCaP cells were transfected and treated as in (**a**). After 30 h of treatment with Tg or DMSO (also transfected with siCtrl), whole cell lysates were prepared and subjected to western blotting with the indicated antibodies; p55/53 Casp8 = procaspase-8, Casp3 = caspase-3 (only cleaved caspase-3 bands are shown), cl-PARP = cleaved PARP. The positions of molecular weight markers are indicated to the left, and nonspecific bands marked with an asterisk. One representative blot out of 4 independent experiments. **c** and **d** Quantifications of the protein levels of cleaved Caspase-3 (**c**) and cleaved PARP (**d**), normalized to the tubulin loading control and then to the Tg + siCtrl condition (mean value set to 1). For (**a**, **c**, and **d**): Mean ± SEM from 4 independent experiments. Dots represent individual values, with a separate color for each experiment. *P < 0.05, **P < 0.01, ***P < 0.001, ns; not significant, One-way ANOVA compared to the Tg + siCtrl condition

ER stress-induced apoptosis has been linked to increased expression of DR5 and activation of caspase-8 in multiple cell types and conditions [9, 13, 17–24], but may also involve DR1 (TNFR1) [26], DR2 (Fas) [27], and DR4 (TRAIL-R1) [19, 20]. To our knowledge, no single study has so far performed a side-by-side comparison of the relative roles played by the various death receptors in ER stress-induced cytotoxicity. To elucidate the putative involvement of different death receptors in Tg-induced cell death in LNCaP and HCT116 cells, we knocked down DR4, DR5 or Fas, or the adaptor proteins FADD and TRADD, which are crucial for cell death induction by death receptors 2, 4, and 5 (FADD) or death receptors 1, 3, and 6 (TRADD), and assessed the effects on Tg-induced cell death. DR5 silencing strongly reduced Tg-induced cell death in both cell lines (Fig. 1a; Additional file 1: Figure S2A), and potently diminished Tg-mediated cleavage of caspase-3 and the caspase-3/7-substrate PARP in both lines (Fig. 1b-d; Additional file 1: Figure S2B, S2D, S2E). Moreover, DR5 depletion virtually abolished Tg-induced caspase-8 cleavage in HCT116 cells (Additional file 1: Figure S2B, S2C). Of note, we could not determine caspase-8 cleavage in LNCaP cells because of too faint bands in the immunoblots (Fig. 1b), in spite of testing a number of different anti-caspase-8 antibodies (not shown). Tg enhanced DR5 protein levels by approximately 3-fold and 9-fold in LNCaP and HCT116 cells, respectively (Additional file 1: Figure S3).

Knockdown of caspase-8 virtually completely blocked Tg-induced cell death as well as caspase-3- and PARP cleavage in both cell lines (Fig. 1; Additional file 1: Figure S2). In contrast, silencing of DR4 or TRADD (see Additional file 1: Figure S4 for knockdown efficiencies) failed to reduce Tg-induced cell death and caspase activation (Fig. 1; Additional file 1: Figure S2). Rather, there was a tendency of a slight increase in Tg-induced cell death and caspase activation in TRADD-silenced LNCaP and HCT116 cells, as well as in DR4-silenced HCT116 cells. These effects did not correlate with any change in DR5 protein expression upon DR4 or TRADD knockdown (Additional file 1: Figure S3). Interestingly, Tg produced a 4- and 2-fold increase in TRADD mRNA levels in LNCaP and HCT116 cells, respectively, and a 2-fold increase in DR4 mRNA levels in HCT116 cells (Additional file 1: Figure S4, B-D), indicating that Tg may limit its own cytotoxicity via these upregulations. Combined knockdown of DR4 and DR5 did not further reduce Tg-induced cell death or caspase activation compared to that obtained upon knockdown of DR5 alone (Fig. 1; Additional file 1: Figure S2). Thus, DR4 was neither required for Tg-induced cell death nor could compensate for the lack of DR5 with regard to Tg-induced cell death, in any of the cell lines.

Tg and other ER stressors have been reported to induce cell death via an intracellular, ligand-independent DR5-nucleated DISC [9, 18]. In agreement, TRAIL-targeted RNAi did not affect Tg-induced cell death (Additional file 1: Figure S5). Although a Tg-induced DR5-nucleated DISC containing FADD and caspase-8 has been demonstrated [9], a specific requirement for FADD in Tg-induced cell death has not been previously examined. In LNCaP cells, FADD silencing efficiently reversed Tg-induced caspase-3 cleavage (Fig. 1b and c), but could only partially reduce Tg-induced cell death and PARP cleavage (Fig. 1a and d). Knockdown of FADD with a second FADD-targeting siRNA likewise only partially reversed Tg-induced cell death (data not shown). In HCT116 cells, FADD knockdown did not significantly alter Tg-induced cell death nor Tg-induced cleavage of caspase-8, caspase-3 or PARP, although there were tendencies of a reduction in Tg-induced caspase-3- and PARP cleavage (Additional file 1: Figure S2). This indicates that proteins other than FADD may contribute to Tg-induced DR5-mediated caspase-8 activation and cell death. Combined knockdown of FADD and TRADD did not reduce Tg-induced cell death or caspase activation compared to that observed upon knockdown of FADD alone (Fig. 1; Additional file 1: Figure S2). Thus, TRADD could not compensate for the lack of FADD. Interestingly, Tg-induced cell death was slightly reduced upon knockdown of Fas in LNCaP cells (Fig. 1a), accompanied by a tendency of a reduction in Tg-induced PARP cleavage (Fig. 1b and d). This effect was not caused by any change in DR5 protein expression upon Fas knockdown (Additional file 1: Figure S3A). The partial requirement for Fas was not observed in HCT116 cells. On the contrary, Fas knockdown slightly increased Tg-induced cell death and PARP cleavage in HCT116 cells (Additional file 1: Figure S2A, S2B, S2E).

In conclusion, our results demonstrate that DR5 and caspase-8 are the main initiators of Tg-induced cell death in both LNCaP and HCT116 cells. Consolidating this conclusion, we confirmed the potent ability of DR5 and caspase-8 silencing to abrogate Tg-induced cell death and caspase activation in both cell lines also with a second set of DR5- and caspase-8-targeting siRNAs (Additional file 1: Figure S6).

### A non-autophagic function of LC3B is partially required for Tg-induced cell death in LNCaP and HCT116 cells

**Fig. 2:**
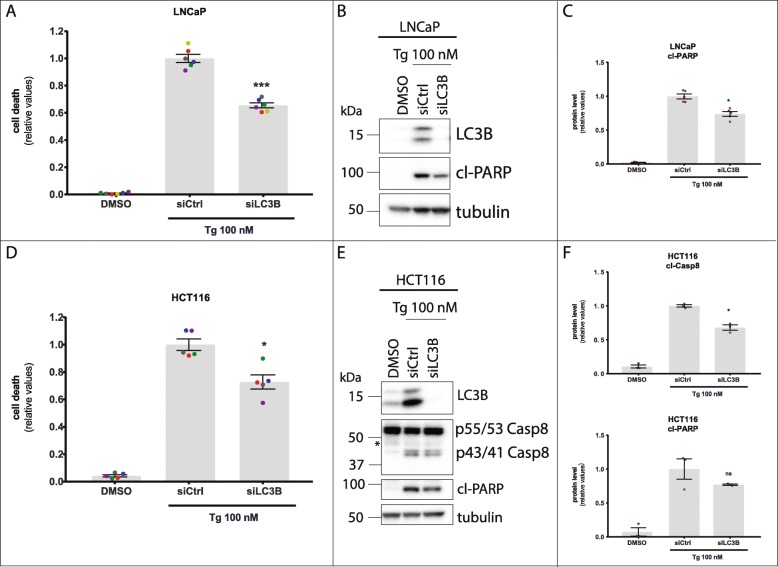
Tg-induced cell death partially requires LC3B. **a** LNCaP cells were transfected for 2 d with the indicated siRNAs (siCtrl = non-targeting control siRNA). Subsequently, cells were treated with 100 nM Tg or 0.01% DMSO vehicle control (“DMSO”, also transfected with siCtrl) in the additional presence of 2.5 μg/ml propidium iodide to stain dead cells. Cell death was monitored and quantified with the IncuCyte ZOOM as described in Materials and Methods, and displayed as relative values normalized to those obtained in the siCtrl+Tg condition after 48 h of treatment (mean value set to 1). Mean ± SEM from 6 independent experiments. **b** LNCaP cells were transfected and treated as in (**a**). After 30 h of treatment with Tg or DMSO (also transfected with siCtrl), whole cell lysates were prepared and subjected to western blotting with the indicated antibodies; cl-PARP = cleaved PARP. The positions of molecular weight markers are indicated to the left. One representative blot out of 4 independent experiments. **c** Quantification of cleaved PARP protein levels, normalized to the tubulin loading control and then to the siCtrl+Tg condition (mean value set to 1). Mean ± SEM from 4 independent experiments. **d** HCT116 cells were transfected and treated as in (**a**), followed by quantification of cell death by propidium iodide staining after 39 h of treatment. Mean ± SEM from 5 independent experiments. **e** HCT116 cells were transfected as in (**d**). After 30 h of treatment with Tg or DMSO (also transfected with siCtrl), whole cell lysates were prepared and subjected to western blotting with the indicated antibodies; p55/53 casp8 = procaspase-8, p43/41 casp8 = cleaved caspase-8, cl-PARP = cleaved PARP. The asterisk indicates a nonspecific band. One representative blot out of 3 independent experiments. **f** Quantification of cleaved caspase-8 bands (upper panel) and cleaved PARP levels (lower panel), normalized to the tubulin loading control and then to the siCtrl+Tg condition (mean value set to 1). Mean ± SEM from 3 independent experiments. For (**a**, **c**, **d**, and **f**): Dots represent individual values, with a separate color for each experiment. *P < 0.05, ***P < 0.001, ns; not significant, One-way ANOVA compared to the Tg + siCtrl condition

The UPR can upregulate the expression of LC3B and other ATGs [29–33], and activate autophagy in LNCaP and other human cell lines [30]. However, due to very efficient ER Ca^2+^ depletion, Tg potently blocks functional autophagy and LC3 flux at a stage prior to phagophore closure, potentially resulting in accumulation of LC3-positive, unclosed autophagosomal membranes [11, 33]. Autophagosomal membranes can act as signaling platforms for oligomerization and activation of caspase-8 in response to treatment with proteasome- or sphingosine kinase inhibitors [63–65]. We therefore hypothesized that Tg-mediated accumulation of LC3-positive autophagosomal membranes may facilitate caspase-8 activation and cell death also in response to Tg-induced ER stress. In line with this hypothesis, knockdown of LC3B led to a ~ 40% reduction of Tg-induced cell death and a ~ 30% reduction of Tg-induced PARP cleavage in LNCaP cells (Fig. 2a-c). Moreover, in HCT116 cells, LC3B silencing led to ~ 30% reduction of Tg-induced cell death, a ~ 30% reduction of Tg-induced procaspase-8 cleavage, and a tendency of a comparable reduction in PARP cleavage (Fig. 2d-f). Similar results were obtained with a second LC3B-targeting siRNA, again with the reduction in Tg-induced cell death being most pronounced in LNCaP cells (Additional file 2: Figure S7A, S7B). Tg strongly increased LC3B protein levels in both LNCaP and HCT116 cells, and LC3B protein expression was efficiently reduced upon LC3B RNAi (Fig. 2b and e; Additional file 2: Figure S7C, S7D). Combined silencing of LC3A and LC3B did not produce any further reduction in Tg-induced cell death compared to that observed upon silencing of LC3B alone (data not shown). Hepatitis B virus-induced autophagy can trigger LC3B-dependent degradation of DR5 [66]. However, and in accordance with Tg blocking functional autophagy [33], knockdown of LC3B did not result in higher DR5 protein levels in Tg-treated cells (Additional file 2: Figure S7, C-F). To test if the requirement for LC3B in Tg-induced cell death reflected a requirement for autophagosomal membranes, we knocked down the ATG genes ATG5 and FIP200, which are crucial for phagophore maturation and recruitment of LC3 to the phagophore [67–69], or all three members of the GABARAP family, which are required for autophagosome formation [70]. Evidently, silencing of ATG5, FIP200, or the GABARAP family members did not reduce Tg-induced cell death in LNCaP or HCT116 cells (Additional file 2: Figure S8A, S8D), in spite of very efficient knockdown (Additional file 2: Figure S8B, S8E). Furthermore, depletion of ATG5, FIP200, or all three GABARAP family members did not result in higher DR5 protein levels in Tg-treated cells (Additional file 2: Figure S8C, S8F), demonstrating - again in accordance with Tg blocking autophagic flux [33] - that DR5 is not subject to autophagic degradation in Tg-treated cells. Together, these results indicate that autophagy and autophagosomal membranes are not required for Tg-induced cell death.

In conclusion, our data indicate that Tg-induced cell death involves a non-autophagic function of LC3B upstream of procaspase-8 cleavage. To our knowledge, this is the first time that LC3 has been implicated as a contributor to cell death induced by a classical ER stressor.

### Tg-induced cell death depends on independent pro-death stimuli from components of the PERK UPR arm, with ATF4 and CHOP, but not PERK, converging on the regulation of DR5 and LC3B protein levels

**Fig. 3:**
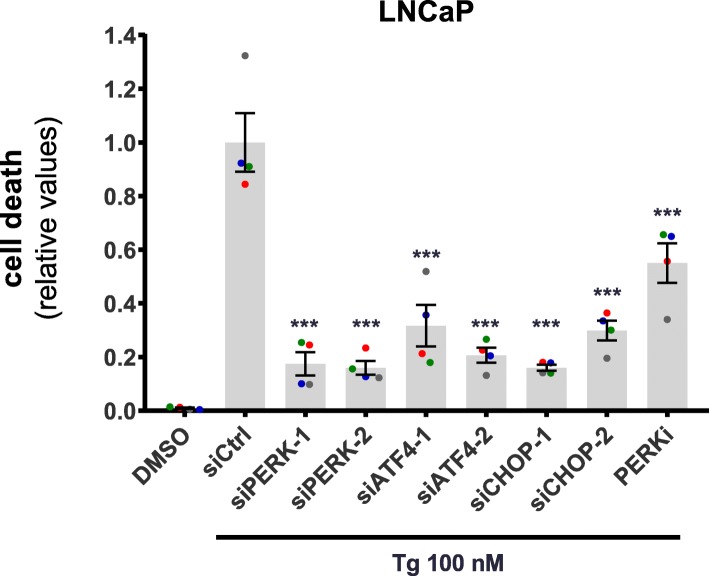
Tg-induced cell death requires PERK, ATF4 and CHOP. LNCaP cells were transfected for 2 d with the indicated siRNAs (siCtrl = non-targeting control siRNA), employing two different siRNA oligoes for each target (designated by − 1 and − 2). Subsequently, cells were treated with 0.02% DMSO (“DMSO”, also transfected with siCtrl) or 100 nM Tg in the absence of presence of 100 nM PERK inhibitor GSK2606414 (PERKi, also transfected with siCtrl), and the additional presence of 2.5 μg/ml propidium iodide in all conditions to stain dead cells. Cell death was monitored and quantified with the IncuCyte ZOOM as described in Materials and Methods, and displayed as relative values normalized to those obtained in the siCtrl+Tg condition after 48 h of treatment (mean value set to 1). Mean ± SEM from 4 independent experiments. Dots represent individual values, with a separate color for each experiment. ***P < 0.001, One-way ANOVA compared to the Tg + siCtrl condition

**Fig. 4:**
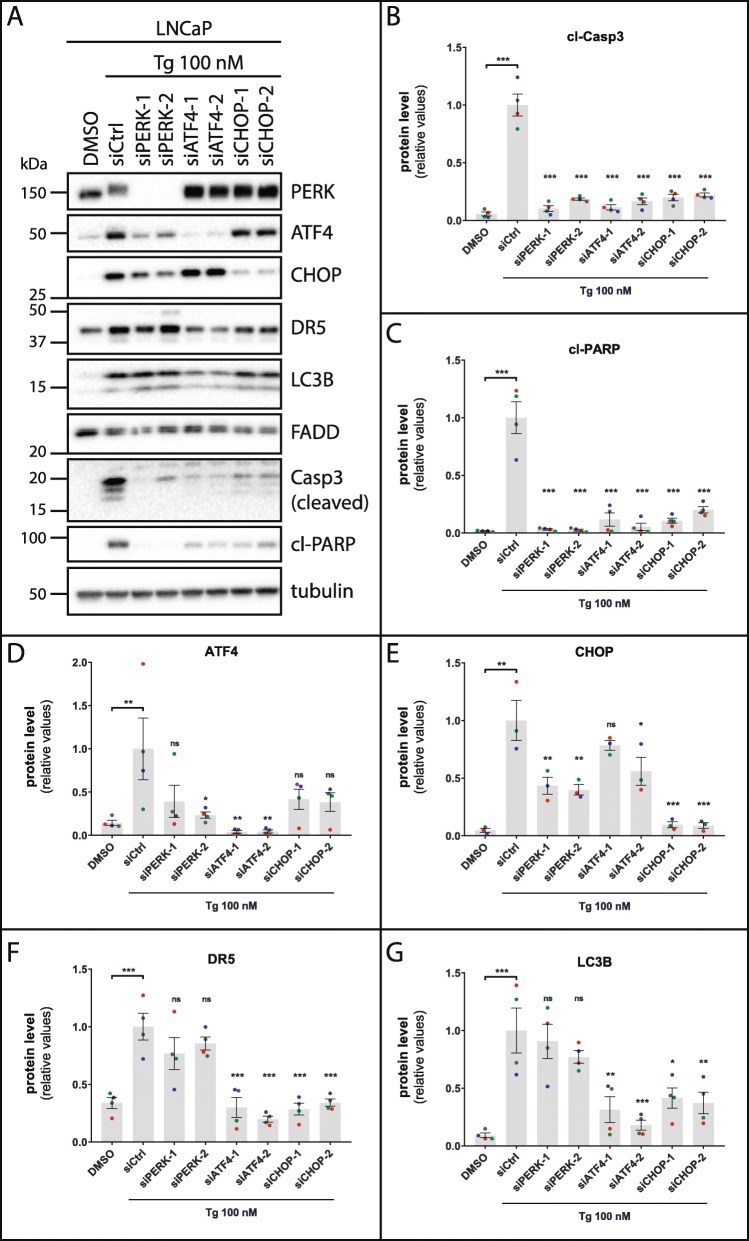
Tg-induced caspase activation requires PERK, ATF4 and CHOP, whereas Tg-mediated upregulation DR5 and LC3B protein levels depends on individual contributions from ATF4 and CHOP, but not PERK. **a** LNCaP cells were transfected for 2 d with the indicated siRNAs (siCtrl = non-targeting control siRNA), employing two different siRNA oligoes for each target (designated by − 1 and − 2). After 30 h of treatment with 100 nM Tg or 0.02% DMSO vehicle control (also transfected with siCtrl), whole cell lysates were prepared and subjected to western blotting with the indicated antibodies; Casp3 = caspase-3 (only cleaved caspase-3 bands are shown), cl-PARP = cleaved PARP. The positions of molecular weight markers are indicated to the left. The shift towards the slower migrating PERK band in Tg-treated cells reflects induction of PERK phosphorylation by Tg. One representative blot out of at least 3 independent experiments. **b-g** Quantifications of western blots from a, normalized to the tubulin loading control and then to the siCtrl+Tg condition. Mean ± SEM from 4 (**b**-**d**, **f**, and **g**) or 3 (**e**) independent experiments. Dots represent individual values, with a separate colour for each experiment. *P < 0.05, **P < 0.01, ***P < 0.001, ns; not significant, One-way ANOVA compared to the Tg + siCtrl condition

Tg-induced apoptosis requires the UPR [8–11, 13]. To enhance our understanding of ER stress-induced cell death initiation, we in the following parts sought to elucidate how the various components of the UPR integrate on the regulation of cell death and the expression of DR5 and LC3B. Knockdown of PERK, ATF4, or CHOP strongly diminished Tg-induced cell death in LNCaP cells (Fig. 3), with comparable reductions in cell death observed with each siRNA and with each target (~ 70–85% reduction). The inhibition of Tg-induced cell death was accompanied by a very strong decrease (~ 80% reduction or more) in Tg-induced cleavage of caspase-3 and PARP (Fig. 4a-c). The dependency on PERK for Tg-induced cell death was likely at least partly due to PERK kinase activity, since treatment with the PERK inhibitor GSK2606414 significantly reduced Tg-induced cell death, albeit to a lesser degree than that observed upon PERK knockdown (Fig. 3). Similar results were obtained in HCT116 cells; knockdown of PERK, ATF4, or CHOP significantly reduced Tg-induced cell death (Additional file 2: Figure S9), as well as the cleavage of caspase-3 and PARP (Additional file 3: Figure S10A-C). Overall, the effects of the knockdowns were more pronounced in LNCaP than in HCT116 cells (Fig. 3; Fig. 4a-c; Additional file 2: Figure S9; Additional file 3: Figure S10A-C) in spite of similar knockdown efficiencies (Fig. 4a, d and e; Additional file 3: Figure S10A, S10D, S10E), indicating that the degree of dependency on the PERK arm for Tg-induced cell death can differ between cell types. The PERK inhibitor significantly reduced Tg-induced cell death also in HCT116 cells (Additional file 2: Figure S9). Taken together, these results demonstrate that Tg-induced cell death substantially depends on PERK, ATF4 and CHOP in both LNCaP and HCT116 cells.

Under UPR-inducing conditions, the PERK-ATF4-CHOP axis is often assumed to operate in a linear fashion, i.e. effects of ATF4 are ascribed to PERK acting via ATF4, and effects of CHOP are ascribed to PERK acting via ATF4 to CHOP. Tg-induced upregulation of ATF4 protein levels did indeed depend on PERK in both LNCaP and HCT116 cells, since it was strongly reduced upon knockdown of PERK at both early (6 h) and late (30 h) time points (6 h: Additional file 4: Figure S11A, S11D; 30 h: Fig. 4a and d; Additional file 3: Figure S10A, S10D). Surprisingly, however, ATF4 silencing had little or no effect on CHOP protein levels at neither of the time points in neither of the cell lines (6 h: Additional file 4: Figure S11A, S11D; 30 h: Fig. 4a and e; Additional file 3: Figure S10A, S10E). Thus, the inhibition of Tg-induced cell death observed upon ATF4 knockdown is very unlikely to be mediated via downregulation of CHOP. Conversely, CHOP silencing, which also diminished Tg-induced cell death, did not significantly alter Tg-induced upregulation of ATF4 protein levels (6 h: Additional file 4: Figure S11A, S11D; 30 h: Fig. 4a and d; Additional file 3: Figure S10A, S10D). We conclude that during Tg-induced cell death in LNCaP and HCT116 cells, ATF4 and CHOP are both required, yet provide their pro-death stimuli independently of each other.

Interestingly, the pro-death roles of ATF4 and CHOP converged in that each of them were individually required for Tg-induced upregulation of DR5 mRNA and protein levels, as well as LC3B mRNA and protein levels, in both LNCaP and HCT116 cells (Fig. 4a, f, and g; Additional file 3: Figure S10A, S10F, S10G; Additional file 4: Figure S11; Additional file 5: Figure S12). In contrast, PERK silencing, which was also required for Tg-induced cell death, failed to significantly alter Tg-mediated upregulation of DR5 and LC3B protein levels (Fig. 4a, f, and g; Additional file 3: Figure S10A, S10F, S10G; Additional file 4: Figure S11A, S11D). Consequently, PERK must be required for an essential Tg-induced pro-death signal that is independent of the regulation of DR5 and LC3B expression, and likely also independent of its regulation of ATF4 and CHOP. Remarkably, PERK silencing did reduce Tg-induced upregulation of DR5 and LC3B at the mRNA level in both cell lines (Additional file 4: Figure S11; Additional file 5: Figure S12). This may be explained by the fact that PERK silencing efficiently diminished Tg-mediated induction of ATF4 in both cell lines, whereas it only partially or transiently reduced Tg-induced upregulation of CHOP in LNCaP and HCT116 cells, respectively (Fig. 4a, d and e; Additional file 3: Figure S10A, S10D, S10E; Additional file 4: Figure S11A, S11D). ATF4 protein upregulation would thus seem to be required for Tg-mediated induction of DR5 and LC3B mRNA expression. The observation that PERK silencing did not block Tg-induced upregulation of DR5 and LC3B protein levels, even though it diminished Tg-mediated induction of ATF4 and Tg-mediated upregulation DR5 and LC3B at the mRNA level, indicates that Tg signals an essential, PERK-independent stimulation of DR5 and LC3B expression at the protein level (protein synthesis and/or stabilization), which does not require ATF4 upregulation. Importantly, unlike PERK silencing, ATF4 knockdown efficiently brought down basal ATF4 protein levels in addition to the levels induced by Tg (Fig. 4a and d; Additional file 3: Figure S10A, S10D). This suggests that basal ATF4 levels are required for Tg-mediated enhancement of DR5 and LC3B at the protein level. Additionally, a threshold level of CHOP seems to be required, since CHOP silencing also blocked Tg-induced upregulation of DR5 and LC3B protein.

### Tg-induced cell death partially requires IRE1 and XBP1 in LNCaP, but not HCT116 cells

**Fig. 5:**
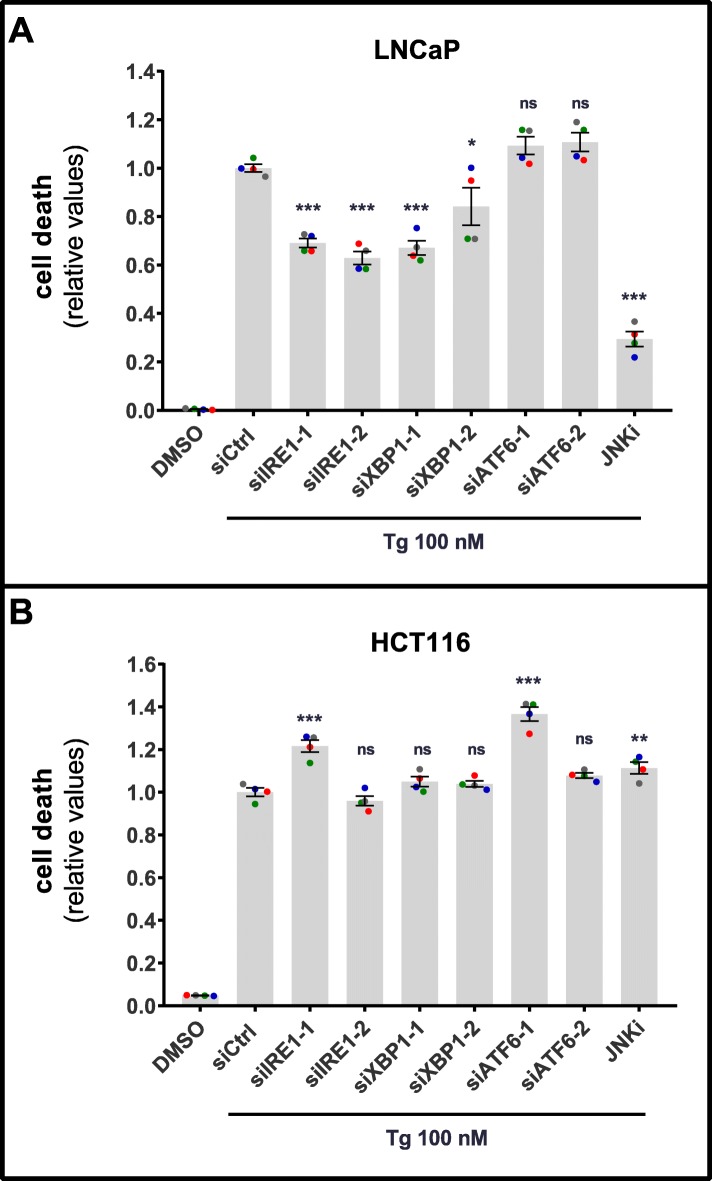
Tg-induced cell death involves IRE1, XBP1, and JNK in LNCaP, but not HCT116 cells (**a** and **b**) LNCaP (**a**) or HCT116 (**b**) cells were transfected for 2 d with the indicated siRNAs (siCtrl = non-targeting control siRNA), employing two different siRNA oligoes for each target (designated by − 1 and − 2). Subsequently, cells were treated with 0.02% DMSO (“DMSO”, also transfected with siCtrl) or 100 nM Tg in the absence of presence of 0.5 μM JNK inhibitor JNK-IN-8 (JNKi, also transfected with siCtrl), and the additional presence of 2.5 μg/ml propidium iodide in all conditions to stain dead cells. Cell death was monitored and quantified with the IncuCyte ZOOM as described in Materials and Methods, and displayed as relative values normalized to those obtained in the siCtrl+Tg condition after 48 h (**a**) or 39 h (**b**) of treatment (mean value set to 1). Mean ± SEM from 4 independent experiments. Dots represent individual values, with a separate color for each experiment. *P < 0.05, **P < 0.01, ***P < 0.001, ns; not significant, One-way ANOVA compared to the Tg + siCtrl condition

**Fig. 6:**
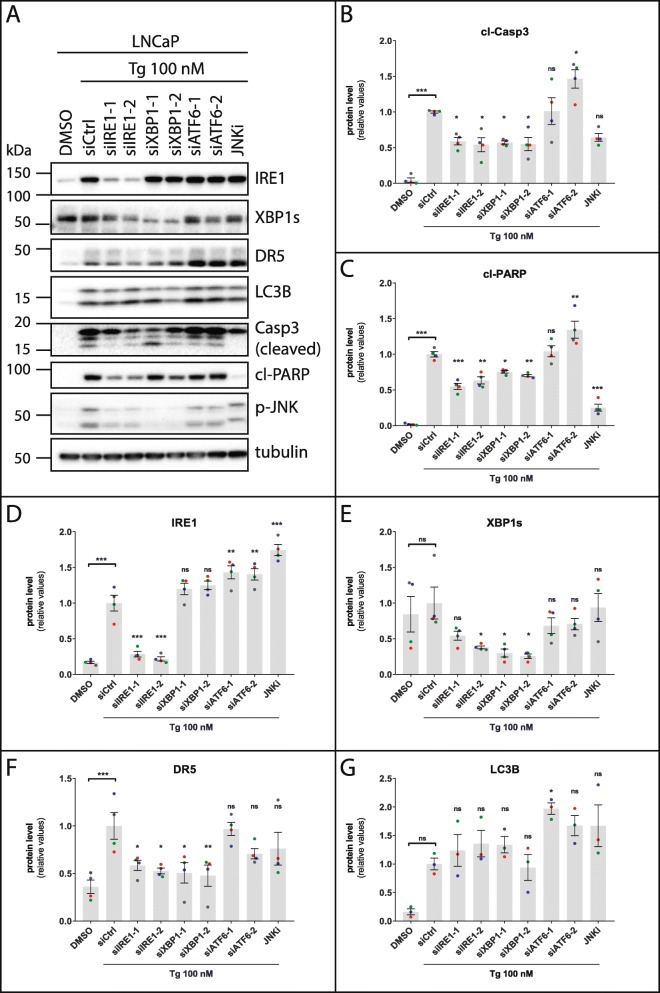
IRE1 and XBP1 are partially required for Tg-induced caspase activation and upregulation of DR5-, but not LC3B expression in LNCaP cells. **a** LNCaP cells were transfected for 2 d with the indicated siRNAs (siCtrl = non-targeting control siRNA), employing two different siRNA oligoes for each target (designated by − 1 and − 2). After 30 h of treatment with 0.02% DMSO (“DMSO”, also transfected with siCtrl) or 100 nM Tg in the absence of presence of 0.5 μM JNK inhibitor JNK-IN-8 (JNKi, also transfected with siCtrl), whole cell lysates were prepared and subjected to western blotting with the indicated antibodies; Casp3 = caspase-3 (only cleaved caspase-3 bands are shown), cl-PARP = cleaved PARP, p-JNK = phospho-JNK. The positions of molecular weight markers are indicated to the left. In the presence of JNK-IN-8, the two p-JNK bands shift towards slightly slower migration due to covalent binding of the inhibitor to the JNK1 and JNK2 isoforms. One representative blot out of at least 3 independent experiments. **b-g** Quantifications of western blots from (**a**), normalized to the tubulin loading control and then to the siCtrl+Tg condition. Mean ± SEM from 4 (b-f) or 3 (**g**) independent experiments. Dots represent individual values, with a separate color for each experiment. *P < 0.05, **P < 0.01, ***P < 0.001, ns; not significant, One-way ANOVA compared to the Tg + siCtrl condition

We next turned to examine the roles played by the IRE1 and ATF6 UPR arms in Tg-induced cell death. In LNCaP cells, knockdown of IRE1 or XBP1 partially reversed Tg-induced cell death, ranging between ~ 20–40% reductions of cell death depending on the siRNA used (Fig. 5a; see Additional file 5: Figure S13A and Fig. 6a, d and e for knockdown efficiencies). This was accompanied by a ~ 30–50% reduction of Tg-induced caspase-3 and PARP cleavage upon IRE1 or XBP1 depletion (Fig. 6a-c). ATF6 silencing did not significantly alter Tg-induced cell death (Fig. 5a; see Additional file 5: Figure S13B for knockdown efficiency). A pro-death role for IRE1 has previously been suggested via signaling to JNK [39, 52–54], and JNK is required for Tg-induced cell death in LNCaP cells [7]. In agreement, the JNK inhibitor JNK-IN-8 efficiently diminished Tg-induced cell death (Fig. 5a). In HCT116 cells, Tg-induced cell death remained the same, or slightly higher, upon knockdown of IRE1, XBP1 or ATF6, or upon JNK-IN-8 co-treatment (Fig. 5b; see Additional file 5: Figure S13C, S13D, and Additional file 5: Figure S14A, S14D, S14E for knockdown efficiencies). The increases in Tg-induced cell death observed with the first siRNAs against IRE1 and ATF6 were not reproducible with a second set of siRNAs (Fig. 5b). Furthermore, there were no significant changes in Tg-induced caspase-3- or PARP cleavage with any of the siRNAs, nor with JNK-IN-8 (Additional file 5: Figure S14 A-C). Together, this indicates that the IRE1 and ATF6 arms do not play any significant role in Tg-induced cell death in HCT116 cells, whereas the IRE1-, but not ATF6 arm is partially required in LNCaP cells.

### The IRE1-XBP1 axis is partially required for Tg-mediated upregulation of DR5-, but not LC3B protein levels

In LNCaP cells, IRE1 or XBP1 silencing resulted in a substantial reduction (~ 60–70% on average) of Tg-induced upregulation of DR5 at the protein level (Fig. 6a and f). In contrast, knockdown of IRE1 or XBP1 did not significantly alter the upregulation of LC3B (Fig. 6a and g). ATF6 silencing or treatment with the JNK inhibitor neither reduced Tg-mediated upregulation of DR5 nor LC3B (Fig. 6a, f, and g). In HCT116 cells, there were no significant alterations of Tg-induced upregulation of either DR5 or LC3B upon knockdown of IRE1, XBP1 or ATF6, nor upon treatment with the JNK inhibitor. Of note, however, there were tendencies of a ~ 40–50% reduction in Tg-induced upregulation of DR5 protein with both XBP1-targeting siRNAs (Additional file 5: Figure S14A, S14F). The reduction in Tg-mediated upregulation of DR5 protein levels upon silencing of the IRE1-XBP1 axis in LNCaP cells may at least in part explain the partial requirement for IRE1 and XBP1 in Tg-induced cell death in this cell line.

### IRE1 does not play an opposing role in Tg-mediated upregulation of DR5 mRNA levels

**Fig. 7:**
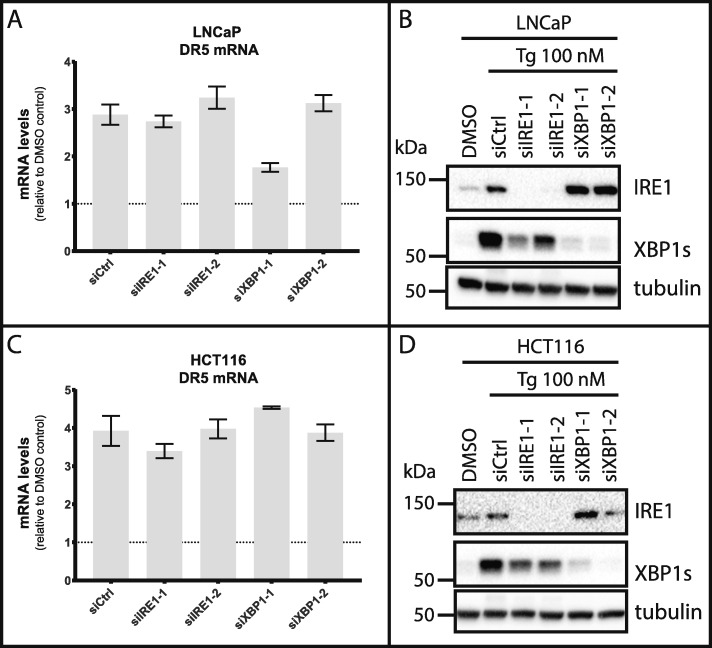
IRE1 does not oppose Tg-induced upregulation of DR5 mRNA. **a-d** LNCaP (**a** and **b**) or HCT116 (**c** and **d**) cells were transfected for 2 d with the indicated siRNAs (siCtrl = non-targeting control siRNA), employing two different siRNA oligoes for each target (designated by − 1 and − 2). Subsequently, cells were treated with 100 nM Tg or 0.01% DMSO (also transfected with siCtrl) for 6 h, and subjected to real-time RT-PCR to quantify DR5 mRNA levels (**a** and **c**) or western blotting to validate efficient knockdown of IRE1 and XBP1s protein levels (**b** and **d**). In (**a** and **c**), relative mRNA levels are shown normalized to the DMSO control-treated condition (set to 1 and indicated by the dotted line in the graphs), i.e. the conditions shown are all with Tg treatment. Mean ± SD of triplicate measurements from one representative experiment out of two. Of note, the reduction observed with siXBP1–1 in (**a**) was not reproducible. In (**b** and **d**), one representative blot out of 2 independent experiments is shown

IRE1 has been reported to induce DR5 mRNA decay via its RIDD activity, and thereby play an opposing role in Tg-induced apoptosis [9]. This activity was reported to occur at relatively early time points after Tg treatment; in the presence of Actinomycin D to block the transcription of new DR5 mRNA, IRE1 silencing was shown to increase DR5 mRNA levels after 4–8 h of treatment with Tg in HCT116 cells [9]. If IRE1-mediated DR5 mRNA decay should play a role in DR5-mediated induction of apoptosis in Tg-treated cells, then IRE1 silencing must result in higher DR5 mRNA levels also in the absence of Actinomycin D. However, this was not examined in the above-mentioned study. To obtain more knowledge on the role of IRE1 in Tg-induced cell death, we tested whether IRE1 knockdown would increase DR5 mRNA levels after 6 h of Tg treatment in LNCaP and HCT116 cells. In spite of efficient silencing, IRE1 knockdown did not lead to any increase in DR5 mRNA levels, in any of the cell lines (Fig. 7). Tg-induced DR5 mRNA levels remained unaltered also upon XBP1 silencing (Fig. 7; the reduction in DR5 mRNA observed with one of the XBP1-targeting siRNAs was neither reproduced by the second XBP1-targeting siRNA nor in a second experiment). Thus, compensatory activation of IRE1 in response to XBP1 depletion [9, 71] did not appear to result in reduced levels of DR5 mRNA. In conclusion, IRE1-mediated DR5 mRNA decay does not seem to have any measurable net effect on DR5 mRNA levels in LNCaP and HCT116 cells.

### The IRE1-XBP1 axis is required for the second phase activation of JNK by Tg

**Fig. 8:**
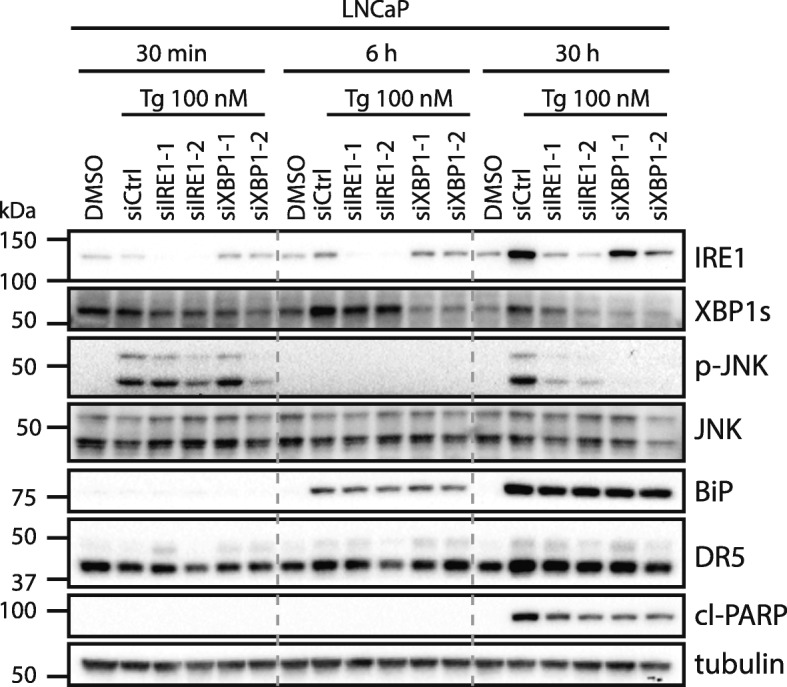
The IRE1-XBP1 axis is required for the second phase of JNK activation by Tg in LNCaP cells. LNCaP cells were transfected for 2 d with the indicated siRNAs (siCtrl = non-targeting control siRNA), employing two different siRNA oligoes for each target (designated by − 1 and − 2). Subsequently, cells were treated with 100 nM Tg or 0.01% DMSO vehicle control (also transfected with siCtrl) for 30 min, 6 h, or 30 h, and subjected to western blotting with the indicated antibodies; p-JNK = phospho-JNK, cl-PARP = cleaved PARP

Tg activates JNK in a biphasic manner in LNCaP cells, with an early activation phase that is discernible after 30 min, yet gone within 6 h, followed by a second activation phase that is discernible from around 12 h and which is sustained at least until 48 h after Tg treatment [7]. At the 30 h time point shown in Fig. 6a, we noted that IRE1 or XBP1 silencing efficiently reversed Tg-mediated upregulation of phospho-JNK levels in LNCaP cells, with the strongest effect observed upon XBP1 knockdown. Similar results were obtained in HCT116 cells (Additional file 5: Figure S14A). This indicates that the IRE1-XBP1 axis is required for the second activation phase of JNK. To determine whether IRE1 and XBP1 would be required also for the first activation phase, we performed a time-course experiment, where we treated IRE1- or XBP1-depleted LNCaP cells with Tg for 30 min, 6 h, or 30 h. Tg increased phospho-JNK levels in the expected biphasic manner (Fig. 8), and in agreement with the above-mentioned results, IRE1 or XBP1 silencing consistently and efficiently reduced Tg-induced upregulation of phospho-JNK at the 30 h time point, again with XBP1 depletion being more effectual than IRE1 depletion. In contrast, IRE1- or XBP1-depletion did not show any consistent reduction of Tg-induced upregulation of phospho-JNK levels at the 30 min time point (Fig. 8), in spite of efficient knockdown and a potent ability of IRE1 depletion to abolish Tg-mediated XBP1s mRNA elevation at 30 min (Fig. 8; Additional file 5: Figure S15). IRE1 and XBP1s thus seem to be required for the second JNK activation phase, but not the first. Of note, the reduction in phospho-JNK levels observed upon IRE1- or XBP1 knockdown was not caused by a reduction in total JNK protein levels (Fig. 8). IRE1 has been reported previously to activate JNK via TRAF2 [39, 40], i.e. via an XBP1-independent IRE1 pathway. If this had been the case in our experiments, we would have expected only IRE1 knockdown and not XBP1 knockdown to abolish Tg-induced JNK activation. Instead, we observed even stronger effects upon XBP1 silencing compared to upon IRE1 silencing. XBP1 knockdown brought down XBP1s mRNA and protein levels more efficiently than IRE1 knockdown (Fig. 6a and e; Fig. 8; Additional file 5: Figure S15C). Thus, the degree of abolishment of Tg-induced upregulation of phospho-JNK levels correlated with the degree of downregulation of XBP1s expression levels. Our results thus suggest that IRE1 acts via XBP1s to regulate the second phase activation of JNK during Tg treatment. Since it is the second, sustained phase of JNK activation that is required for Tg-induced apoptosis [7, 72], the involvement of IRE1-XBP1 in this phase can at least partly explain the requirement for the IRE1-XBP1 axis in Tg-induced cell death of LNCaP cells.

### Cell death induced by therapeutically relevant Tg analogs display the same requirements for DR5, UPR components, and JNK as cell death induced by Tg

To optimize the use of Tg prodrugs in cancer therapy, we need to know if the Tg analogs that are produced upon prodrug cleavage in vivo display the same mechanisms of action as the mother compound Tg. The prodrugs that have been tested and shown promising results in pre-clinical and clinical trials contain peptide sequences that upon cleavage produce the Tg analogs Leu-8ADT [14, 57, 58] or βAsp-8ADT [15, 59, 73] (for structures see Additional file 1: Figure S1A). These analogs display a slower binding to SERCA than Tg, but just like Tg they efficiently deplete ER Ca^2+^, activate a sustained UPR, and induce apoptotic cell death in cancer cells [10]. However, it is not further known whether Tg analogs and Tg induce cell death via the same molecular mechanisms or not. Now that we had elucidated many of the key molecular requirements for Tg-induced cell death, and noting the slower kinetics of the therapy-relevant analogs for SERCA inhibition, we were curious to compare their effects to those of Tg in more detail. To that end, we put Leu-8ADT and βAsp-8ADT through the same focused RNAi screen as we had done with Tg, and assessed the effects on cell death in both LNCaP and HCT116 cells. Strikingly, Leu-8ADT and βAsp-8ADT both showed virtually identical molecular requirements for cell death induction as those we had observed with Tg, in both cell lines. Thus, Leu-8ADT- and βAsp-8ADT-induced cell death showed a strong requirement for caspase-8 and DR5, but not DR4, in both LNCaP and HCT116 cells (Additional file 5: Figure S16). Furthermore, just like with Tg, cell death partially required FADD in LNCaP cells, whereas the effect of FADD knockdown was weak in HCT116 cells. Also, with Leu-8ADT and βAsp-8ADT there was an overall trend of a further increase in cell death upon TRADD knockdown, and while Fas knockdown produced a slight increase in cell death in HCT116 cells, Fas was partially required for Leu-8ADT- and βAsp-8ADT-induced cell death in LNCaP cells (Additional file 5: Figure S16). PERK, ATF4, and CHOP were all required for Leu-8ADT- and βAsp-8ADT-induced cell death in both cell lines, with the strongest requirement observed in LNCaP (Additional file 5: Figure S17), i.e. exactly like we had observed with Tg. Lastly, while ATF6 depletion did not alter cell death in any of the cell lines, IRE1, XBP1, and JNK were partially required for Leu-8ADT- and βAsp-8ADT-induced cell death in LNCaP, but not HCT116 cells (Additional file 5: Figure S18), again as we had observed with Tg. In conclusion, these results indicate that therapeutically relevant Tg analogs induce the same key molecular pro-death stimuli as Tg. Mechanistic investigations of the cytotoxic actions of Tg can thus be expected to have important value not only for our understanding of UPR-induced cell death but also for our understanding and potential to optimize the therapeutic use of Tg prodrugs.

## Discussion

Understanding the molecular mechanisms of Tg-induced cell death is important for enhancing our knowledge of ER stress-induced apoptosis, opening up for the development of novel treatment and combination treatment strategies towards diseases that are associated with lowered ER Ca^2+^ levels, chronic ER stress and cell death [2–4], as well as in Tg-based targeted cancer therapy [16]. Although much is known about downstream cell death-inducing mechanisms in response to unmitigated ER stress, e.g. the involvement of caspases and various Bcl-2 protein family members [5, 74, 75], the initiating events - and how the various components of the UPR integrate on them - are less well defined. Moreover, the role played by autophagy and ATG proteins, which are very often implicated in cell fate decisions [28], is uncertain.

Numerous studies have shown that ER stress-induced cell death-initiation involves death receptor-associated activation of caspase-8 [9, 13, 17–22, 26, 27]. However, it is unclear whether one member of the death receptor family is more important than others, or whether ER stress-induced cell death may generally co-require several different death receptors. DR5 has been shown to play a prominent role in ER stress-induced cell death [9, 13, 18, 21], but other studies have indicated the involvement of DR4 [19], TNFR1 [26], or Fas [27]. Direct comparison has been made only with regard to the related DR4 and DR5. DR4 and DR5 knockout cell lines were shown to display comparable deficiencies in Tg-induced cell death, suggesting that DR4 and DR5 contribute equally to ER stress-induced cell death [20]. Recent studies have however demonstrated that caution must be exerted with regard to drawing strong conclusions from death receptor knockout studies [21, 76]. Thus, whereas Tg-induced apoptosis was only minimally or partially abolished in DR5 HCT116 knockout cells [21, 76] and could not be further reduced by either DR5- or caspase-8-targeting siRNAs [21], Tg-induced cell death was nearly completely abolished by siRNA-mediated depletion of DR5 or caspase-8 in the parental cell line [21]. This indicates that the long-term DR5 ablation in the knockout cell line had caused adaptation to a rewired ER stress- responsive apoptotic pathway [21]. More acute depletion methods may thus be more appropriate than permanent knockouts to elucidate the endogenous roles of the various death receptors in ER stress-induced cell death. Using an RNAi-based approach, we showed that Tg-induced cell death depended strongly on DR5 and caspase-8 in both LNCaP and HCT116 cells, whereas DR4 was not required in either cell line, indicating a dominant role of DR5 versus DR4 in ER stress-induced cell death. In line with this, Tg was previously shown to upregulate DR5 but not DR4 mRNA expression in 10 different cell lines [17], and another study showed that Tg potently upregulated DR5, but elicited little or no increase in DR4, TNFR1, or Fas mRNA expression in two different cell lines [9]. It should be noted that those studies examined neither the protein expression levels nor the potential requirement for DR4, TNFR1, or Fas in ER stress-induced apoptosis. A recent study found that glucose deprivation (which induces many forms of cellular stress, including ER stress) could increase DR4 protein levels without increasing DR4 mRNA levels in HeLa cells, and glucose deprivation-induced cell death showed comparable dependencies on DR4 and DR5 [77]. Interestingly, our results showed that Fas was partially required for Tg-induced cell death in LNCaP, but not HCT116 cells, suggesting a co-requirement for DR5 and Fas in ER stress-induced cell death in LNCaP cells (yet with the strongest dependency on DR5). TRADD was not required for Tg-induced cell death in any of the cell lines, suggesting that TNFR1 and members of the DR3 and DR6 family are not involved. In fact, TRADD silencing tended to increase rather than decrease Tg-mediated cell death and caspase activation. This finding may be related to previous observations showing that TRADD can protect cells from TRAIL-induced apoptosis [78, 79], in part via competition with FADD for binding to the DR4/DR5 receptor complex [78]. However, given that ligand-independent death receptor complexes may differ in their requirement for adaptor proteins (see further discussion below), our data cannot formally exclude a potential TRADD-independent pro-death involvement of DR1, DR3 or DR6 members in Tg-induced cell death. In any case, the severe reduction in Tg-induced cell death that we observed upon knockdown of DR5 in both LNCaP and HCT116 indicates a major requirement for DR5. In conclusion, although other death receptors may also play a contributing role, our results and those of others [9, 13, 17, 18, 20, 21] strongly suggest that DR5 is a main hub for ER stress-mediated initiation of cell death across a range of different cell types.

Our observation that TRAIL-targeting RNAi did not alter Tg-induced cell death is in line with previous studies showing that Tg and other ER stressors can trigger DR5-dependent apoptosis independently of the DR5 ligand (TRAIL) [9, 18]. Interestingly, Tg-induced DR5 multimerization and caspase-8 activation most likely occur at intracellular sites [9]. It is not known whether caspase-8 activation by this ER stress-induced, ligand-independent intracellular DR5 involves the same DISC components as classical ligand-activated plasma membrane DISCs. Fas can undergo ligand-independent multimerization, leading to FADD recruitment and caspase-8 activation in response to UV light or anticancer drugs, but under those conditions the death receptor aggregation occurs at the plasma membrane, and not intracellularly [80, 81]. Tg was shown to induce DR5-FADD-caspase-8 complexation in HCT116 cells [9], but it was not examined if, or to which degree, this occurred at the intracellular sites of DR5 multimerization. The requirement for FADD in Tg-induced cell death and caspase activation has not been examined previously. Surprisingly, we observed that, unlike DR5 knockdown, FADD knockdown did not significantly reduce Tg-induced caspase-8 cleavage or cell death in HCT116 cells. Moreover, in LNCaP cells, FADD depletion had a markedly weaker effect on Tg-induced cell death and caspase activation than that observed upon DR5- or caspase-8 depletion. This may suggest that even very low amounts of FADD are sufficient to mediate caspase-8 recruitment to DR5 under ER stress conditions. However, RNAi-mediated reduction of FADD protein levels is reportedly sufficient to abolish TRAIL- and Fas-mediated caspase activation and cell death [82, 83]. Taken together, our data, therefore, more likely indicate that ER stress-induced DR5-dependent intracellular activation of caspase-8 and cell death involves adaptor proteins other than FADD, in particular in HCT116 cells. In line with this, a previous study showed that apoptosis induced by overexpression of DR5 (i.e. without ligand present) in HeLa cells was not inhibited by the expression of a dominant negative form of FADD [84].

The current study is, to the best of our knowledge, the first to identify a role for LC3B in ER stress-induced cell death. Specifically, LC3B was partially required for caspase-8 activation and cell death in Tg-treated cells. LC3B is best known for its role in autophagy. When attached to the phagophore (the autophagosomal membrane that matures into the autophagosome), LC3B acts to recruit autophagic cargo via binding to adaptor proteins called autophagy receptors, e.g. SQSTM1 (p62) [85]. The phagophore contains a number of ATGs, including FIP200 and ATG5, which are required for LC3 recruitment and phagophore maturation [67–69]. The LC3-related GABARAP protein family is also essential for autophagosome formation, but not for LC3 recruitment [70]. Previous studies have indicated that autophagosomal membranes can serve as platforms for intracellular caspase-8 activation in response to treatment with proteasome or sphingosine kinase inhibitors [63–65]. Specifically, recruitment of caspase-8 to autophagic membranes was suggested to occur via ATG5-FADD-caspase-8 [63, 65] and/or LC3-p62-caspase-8 [64, 65] complexes. In our experiments, knockdown of FIP200, ATG5, or the GABARAPs all failed to reduce Tg-induced cell death. This indicates that autophagosomal membranes are unlikely to serve as platforms for Tg-induced caspase-8 activation and that a non-autophagic function of LC3B is involved instead. Of note, LC3B can interact with p62 also when not present on autophagosomal membranes [86, 87]. Thus, a putative Tg-induced LC3B-p62-caspase-8 interaction could potentially explain our observations. However, p62 depletion did not reduce Tg-induced cell death (data not shown), thus implicating the involvement of other, yet to be defined mechanisms of LC3B-dependent caspase-8 activation during ER stress.

**Fig. 9:**
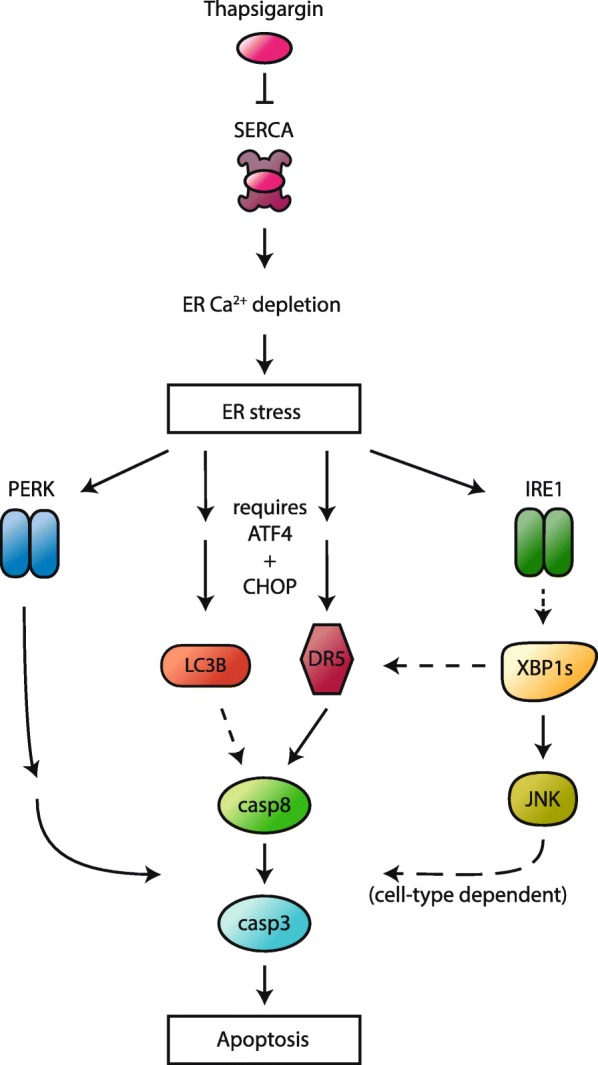
Schematic depiction of main conclusions. Thapsigargin (Tg) binds to and blocks SERCA (the ER Ca^2+^ pump), thus causing ER Ca^2+^ depletion and ER stress. ER stress upregulates LC3B and DR5 protein levels in a manner that requires individual contributions from ATF4 and CHOP. Tg-induced activation of caspase-8 (casp8), caspase-3 (casp3), and apoptosis is strongly dependent on DR5, and partially dependent on LC3B. PERK is required for Tg-induced caspase activation and apoptosis, but not for upregulation of LC3B- and DR5 protein levels. Thus, PERK delivers an essential pro-death signal via a different pathway. The IRE1-XBP1 axis is partially required for Tg-induced upregulation of DR5, but not LC3B. Moreover, IRE1-XBP1 is required for the second-phase activation of JNK by Tg, which contributes to caspase activation and apoptosis in a cell type-dependent manner with JNK being required for Tg-induced cell death in LNCaP but not HCT116 cells

Tg and other ER stressors are known to induce cell death via the UPR, but the contributions and integration of the individual UPR components to cell death and its initiation remain incompletely defined. In order to address these important questions, we have in the current study systematically examined how each of the central UPR components (PERK, ATF4, CHOP, IRE1, XBP1, and ATF6) regulates Tg-induced caspase activation and cell death in LNCaP and HCT116 cells. Moreover, to gain more insight into UPR-mediated regulation of cell death initiation, and having identified DR5 and LC3B as important initiating factors of Tg-induced cell death in both cell lines, we simultaneously delineated how the various UPR components regulate DR5- and LC3B expression. Previous studies on the role of the UPR arms in ER stress-induced cell death and DR5 expression have examined either only one cell line (leaving uncertainty regarding potential cell type-specific effects) or only a subset of UPR components (leaving uncertainty about the wider picture), using only one siRNA per target (leaving uncertainty to potentially confounding off-target effects). To overcome these limitations, we set out to target each of the main UPR components with two different siRNAs per target in two different cell lines and assess the effects on Tg-induced cell death, caspase activity, and DR5- and LC3B expression. We found that the PERK arm components PERK, ATF4, and CHOP were all required for Tg-induced cell death in both LNCaP and HCT116 cells, yet acted in parallel rather than as a linear pathway. CHOP has been found to be required for ER stress-induced DR5 upregulation and cell death in several studies [9, 13, 18], and this has been assumed to be the result of CHOP acting downstream of the PERK-ATF4 axis [9, 18, 88, 89]. Importantly, however, this assumption has not been experimentally tested. Remarkably, we observed that ATF4 depletion failed to abolish Tg-mediated upregulation of CHOP, and we could, therefore, conclude that ATF4 and CHOP provide their pro-death stimuli independently of each other. Interestingly, the roles of ATF4 and CHOP converged in that each of them was essential for Tg-mediated upregulation of DR5- and LC3B expression at both the mRNA and protein levels. PERK depletion abolished Tg-mediated upregulation of ATF4 protein expression and reduced Tg-mediated upregulation of DR5- and LC3B mRNA levels, but surprisingly, and in contrast to the effect of ATF4 or CHOP silencing, it failed to significantly reduce Tg-mediated upregulation of DR5 and LC3B at the protein level. Unlike PERK silencing, which merely abrogated Tg-mediated ATF4 upregulation, ATF4 silencing in Tg-treated cells depleted ATF4 protein levels to well below the basal levels found in DMSO control-treated cells. This indicates that PERK-dependent upregulation of ATF4 is required for Tg to increase DR5- and LC3B mRNA expression, whereas Tg-mediated increase in DR5- and LC3B protein expression requires basal ATF4 levels, but not PERK. Tg-mediated upregulation of DR5 and LC3B mRNA and protein levels also requires an independent contribution from CHOP. Together, our findings point to a cell death-essential, Tg-induced regulation of DR5 and LC3B at the protein level (enhanced protein synthesis and/or increased protein stabilization), which is dependent on ATF4 and CHOP, but not on PERK. The specific regulation of DR5 and LC3B at the protein level may be equally or even more important than their regulation at the mRNA level. An exclusive regulation of DR5 at the protein level has been observed in response to glucose deprivation of HeLa cells, where DR5 protein levels rose in the 24–72 h time period in the absence of any increase in DR5 mRNA levels [77]. Under those conditions, the upregulation of DR5 protein levels was abolished upon silencing of ATF4, but not CHOP [77], whereas the putative role of PERK or other kinases of the integrated stress response was not examined. The role of CHOP in DR5 regulation may thus differ depending on the stress condition. Regulation of LC3B at the protein level has not been much studied, but interestingly, it has been shown that T cell activation increases LC3B protein expression exclusively at the translational level [90]. In summary, our delineation of the roles played by the components of the PERK UPR arm indicates that Tg-induced ER stress triggers cell death via ATF4- and CHOP-dependent upregulation of DR5 and LC3B protein expression, as well as via other mechanisms, which require PERK (Fig. 9).

Interestingly, we observed a cell type-dependent role of IRE1 in ER stress-induced cell death; whereas IRE1 was partially required for Tg-mediated cytotoxicity in LNCaP cells, its depletion did not significantly alter Tg-induced cell death in HCT116 cells. Mechanistically, we could link the pro-death role of IRE1 to a previously unrecognized XBP1-dependent activation of JNK. JNK initiates a complex and varied set of intracellular signals that affect cell death and survival in a context- and cell type-dependent manner [91]. Intriguingly, although Tg increased phospho-JNK levels in an IRE1/XBP1-dependent manner in both cell lines, JNK was selectively required for Tg-induced cell death in LNCaP, and not in HCT116 cells. Together, these results provide an explanation to the observed cell type-dependent role of IRE1 in that the IRE1-XBP1-JNK pathway contributes to ER stress-induced cell death only in cell types that are sensitive to the pro-death effects of JNK (Fig. 9). This may at least in part explain why IRE1 is found to be required for ER stress-induced cell death in some cell types [49, 53, 54] while having no effect on cell death in others [18, 92–94]. We could not observe any pro-survival effect of IRE1 or XBP1 during Tg-induced cell death in the cell lines we examined. Moreover, and in contrast to what has been suggested in HCT116 cells previously [9], we could not observe any opposing role of IRE1 on Tg-mediated upregulation of DR5 mRNA in HCT116 or LNCaP cells. Thus, IRE1-activated RIDD of DR5 mRNA does not appear to be a general pro-survival mechanism for IRE1, and IRE1 does not appear to play a general opposing role during ER stress-induced cell death.

The IRE1-XBP1 axis has been widely described to provide adaptive and anti-apoptotic signals during ER stress [47–49, 74, 95–98]. A current model proposes that XBP1-mediated pro-survival signals pre-dominate over other IRE1-mediated effects during mild, resolvable ER stress, whereas IRE1 RIDD- and IRE1-TRAF2-JNK pro-death signals override the XBP1-mediated pro-survival signals during cytotoxic, unresolvable ER stress [49, 74, 96, 97]. Importantly, however, the exact role played by the IRE1-XBP1 axis during cytotoxic ER stress has not been directly experimentally tested previously. Our results in LNCaP cells provide the first direct demonstration of a pro-death role for XBP1 during cytotoxic ER stress conditions. In line with this finding, two previous studies have shown that high levels of XBP1s can mediate pro-apoptotic signals. Firstly, it was shown that overexpression of XBP1s induced apoptosis in human umbilical endothelial cells [99]. Secondly, a recent report showed that while XBP1 mediated adaptive signals at non-cytotoxic, low levels of ER stress, it could mediate pro-apoptotic effects via upregulating the expression of the transcription factor KLF9 at cytotoxic, high levels of ER stress in WI38 human fibroblasts [100]. Although the effect of XBP1 depletion on ER stress-induced cell death was not examined in the latter report, these studies and ours together suggest that the IRE1-XBP1 axis provides pro-death signals under high-level ER stress conditions and that this may contribute to ER stress-induced cell death in several different cell types. By demonstrating that Tg-induced JNK-activation depends on the IRE1-XBP1 axis, we have identified a novel path for ER stress-induced pro-death signals. Previously, IRE1 was shown to activate JNK via direct interaction between IRE1 and TRAF2 [39, 40]. Overexpression studies with IRE1β in HEK293T cells indicated that the IRE1-TRAF2 interaction requires the IRE1 kinase-, but not RNAse activity [40], i.e. that it is independent of the IRE1-XBP1 axis. Consequently, although we cannot exclude that XBP1 may be required for the IRE1-TRAF2-JNK axis in a cell type-dependent manner (i.e. in LNCaP and HCT116, but not HEK293T cells), we consider it most likely that our results reflect a novel mechanism of JNK activation during unresolvable ER stress. Interestingly, Tg enhances JNK activity in two distinct temporal phases consisting of a rapid and transient phase that provides pro-survival signals [72], followed by a second, sustained phase that mediates pro-death signals [7]. The molecular mechanisms responsible for JNK activation in the first versus the second phase may be different. Whereas the IRE1-TRAF2-JNK pathway, which is activated very rapidly (within 10–30 min) after Tg stimulation [39, 40, 72], drives the first phase of JNK activation, the mechanism responsible for the second phase, sustained activation of JNK is unknown. Our results indicate that, at least in LNCaP and HCT116 cells, the second phase activation of JNK is mediated by an IRE1-XBP1-dependent mechanism. It is important to note that although the IRE1-TRAF2-ASK1-JNK pathway is well documented [39, 52, 101], direct and conclusive evidence for its role in mediating ER stress-induced cell death is lacking. For example, Tg failed to induce apoptosis in ASK1 knockout MEFs [39], but the role of IRE1, TRAF2, and JNK were not examined. More detailed evidence has been demonstrated in studies of the effects of RNF13 or Bak + Bim/PUMA overexpression [52, 101], but it is unclear how those conditions compare with general ER stress-inducing conditions. Thus, the roles of the IRE1-TRAF2-ASK1-JNK- and the herein defined IRE1-XBP1-JNK pathways in ER stress-induced cell death should be carefully examined in future studies. In addition to IRE1-XBP1-dependent regulation of JNK, we found that IRE1 and XBP1 were partially required for Tg-induced upregulation of DR5 protein in LNCaP cells. This was not mediated by JNK since the JNK inhibitor did not affect DR5 protein levels. Moreover, interference with IRE1, XBP1, or JNK did not affect the levels of LC3B in Tg-treated cells. In summary, the requirement for IRE1 and XBP1 in Tg-induced cell death in LNCaP cells may in part be explained by IRE1-XBP1-dependent regulation of JNK and in part by IRE1-XBP1-dependent regulation of DR5 protein levels (Fig. 9).

Lastly, we found that the two therapeutically relevant Tg analogs Leu-8ADT and βAsp-8ADT induced cell death with virtually identical molecular requirements as those observed for Tg in both LNCaP and HCT116 cells. These are important findings that could not be taken for granted, since the analogs differ from Tg by the addition of a 12-aminododecanoyl side-chain (in position O-8), to which an amino acid residue (either a leucine or an aspartate residue) resulting from proteolysis-mediated prodrug peptide cleavage, is attached. Compared to Tg, the analogs show differences in their interaction with SERCA (burial of the extended prodrug linker) and display a slower binding to SERCA, even though the analogs bind to the same binding site [10, 15, 102, 103]. The differences result in higher concentrations being needed for cell death induction [10]. Interestingly, however, Leu-8ADT is, for reasons that still need to be fully elucidated [10], a more potent killer than βAsp-8ADT. Across 3 different cancer cell lines, 50% of maximal cell death induction was reached at 1.4- to 4.6-fold higher concentrations of Leu-8ADT than Tg, whereas 5- to 13-fold higher concentrations than Tg were required for βAsp-8ADT [10]. In spite of these differences, our current results indicate that when cancer cells are exposed to cytotoxic concentrations of Leu-8ADT or βAsp-8ADT they are killed via the same mechanisms as when they are exposed to Tg. This is encouraging and vital information for future use and optimization of Tg prodrugs in cancer treatment, including in combination therapy. With regard to the latter, the βAsp-8ADT-producing Tg prodrug has been successfully combined with the antiangiogenic drug tasquinimod to completely eradicate tumor growth in breast cancer xenografts [104]. Compared to conventional chemotherapy, Tg has the advantage of efficiently killing tumor cells independently of their proliferation rate [105]. Additionally, Tg blocks autophagy [33], thereby avoiding the putative pro-survival effects from autophagic activity that are often observed in response to therapy, and which may contribute to therapeutic resistance [106]. Of note, the Tg analogs paralleled the effect of Tg in efficiently inhibiting autophagic activity (data not shown). Thus, Tg prodrugs may find use in combination with therapeutic treatments that otherwise induce protective autophagy. The Tg-mediated block in autophagic flux likely increases the ability of Tg and the analogs to induce cell death since the consequential inhibition of autolysosomal LC3B degradation increases the protein levels of this contributing factor to ER stress-induced apoptosis.

## Conclusion

In conclusion, our systematic study has provided important new insights into both general and cell type-specific signaling mechanisms of cell death-induction upon Tg-induced ER stress, thus contributing to increase our understanding of ER stress-induced cell death and providing knowledge that is of relevance to diseases associated with UPR-induced cell death, as well as to Tg-based cancer therapy.

## Supplementary information

**Additional file 1: Figure S1.** Chemical structures of drugs used in the current study. **(a)** Tg and the Tg analogs 8-O-debutanoyl-8-O-N-L-Leucinoyl-12-aminododecanoylthapsigargin (Leu-8ADT) and 8-O-debutanoyl-8-O-N-L-b-aspartoyl-12-aminododecanoylthapsigargin (βAsp-8ADT) **(b)** The PERK inhibitor GSK2606414 **(c)** the JNK inhibitor JNK-IN-8. **Figure S2.** Tg-induced cell death depends on DR5 and caspase-8, but not DR4, Fas, FADD, or TRADD in HCT116 cells. **Figure S3.** Quantification of DR5 protein levels (related to Fig. 1 and Fig. S2). **Figure S4.** DR4, TRADD and Fas knockdown confirmations (related to Fig. 1 and Fig. S2). **Figure S5.** Tg-induced cell death does not require TRAIL. **Figure S6.** DR5 and caspase-8 are strongly required for Tg-induced cell in both LNCaP and HCT116 cells (related to Fig. 1 and Fig. S2).

**Additional file 2: Figure S7.** LC3B is partially required for Tg-induced cell death, and LC3B depletion does not increase DR5 protein levels. **Figure S8.** Tg-induced cell death does not require the key autophagic membrane components ATG5, FIP200, or GABARAPs. **Figure S9.** Tg-induced cell death depends on PERK, ATF4 and CHOP in HCT116 cells.

**Additional file 3: Figure S10.** Tg-induced caspase activation in HCT116 cells requires PERK, ATF4 and CHOP, whereas Tg-mediated upregulation DR5- and LC3B protein levels depends on individual contributions from ATF4 and CHOP, but not PERK.

**Additional file 4: Figure S11.** Regulation of Tg-mediated upregulation of DR5- and LC3B protein and mRNA levels by PERK, ATF4 and CHOP at an early time point (6 h).

**Additional file 5: Figure S12.** Tg-mediated upregulation of DR5- and LC3B mRNA levels requires PERK, ATF4 and CHOP in LNCaP and HCT116 cells. **Figure S13.** IRE1 and ATF6 knockdown confirmations (related to Fig. 5). **Figure S14.** Tg-mediated caspase activation and upregulation of DR5 and LC3B does not require IRE1, XBP1, ATF6, or JNK in HCT116 cells. **Figure S15.** Tg rapidly enhances XBP1s mRNA levels in an IRE1-dependent manner in LNCaP cells (related to Fig. 8). **Figure S16.** Cell death induced by the therapeutically relevant Tg analogs Leu-8ADT and βAsp-8ADT requires DR5 and caspase-8 in LNCaP and HCT116 cells, and partially requires FADD and Fas in LNCaP cells, whereas DR4 and TRADD are not required in any of the cell lines. **Figure S17**. Cell death induced by Leu-8ADT and βAsp-8ADT requires PERK, ATF4, and CHOP in LNCaP and HCT116 cells. **Figure S18.** Cell death induced by Leu-8ADT and βAsp-8ADT involves IRE1, XBP1, and JNK in LNCaP, but not HCT116 cells.

## Data Availability

All data generated or analysed during this study are included in this published article and its additional information files.

## Abbreviations

ANOVA: analysis of variance
ATG: autophagy-related
DISC: death-inducing signaling complex
DR: death receptor
ER: endoplasmic reticulum
JNK: c-Jun N-terminal kinase
LC3B: microtubule-associated proteins 1A/1B light chain 3B (MAP1LC3B)
Leu-8ADT: 8-*O*-debutanoyl-8-*O*-*N*-L-Leucinoyl-12-aminododecanoylthapsigargin
MEFs: mouse embryonic fibroblasts
RIDD: Regulated IRE1-dependent decay
SEM: standard error of the mean
SERCA: sarco/endoplasmic reticulum Ca^2+^-ATPase
Tg: thapsigargin
Tm: tunicamycin
UPR: unfolded protein response
XBP1s: spliced XBP1
βAsp-8ADT: 8-*O*-debutanoyl-8-*O*-*N*-L-β-aspartoyl-12-aminododecanoylthapsigargin
