# Supplementary material for: Cell death induced by the ER stressor thapsigargin involves death receptor 5, a non-autophagic function of MAP1LC3B, and distinct contributions from unfolded protein response components
Source: Cell Commun Signal. 2020 Jan 27;18:12. doi: 10.1186/s12964-019-0499-z (PMC6986015; doi:10.1186/s12964-019-0499-z)
Supplement: Supplementary file 2 — Additional file 1: Figure S1. Chemical structures of drugs used in the current study. (a) Tg and the Tg analogs 8-O-debutanoyl-8-O-N-L-Leucinoyl-12-aminododecanoylthapsigargin (Leu-8ADT) and 8-O-debutanoyl-8-O-N-L-b-aspartoyl-12-aminododecanoylthapsigargin (βAsp-8ADT) (b) The PERK inhibitor GSK2606414 (c) the JNK inhibitor JNK-IN-8. Figure S2. Tg-induced cell death depends on DR5 and caspase-8, but not DR4, Fas, FADD, or TRADD in HCT116 cells. Figure S3. Quantification of DR5 protein levels (related to Fig. 1 and Fig. S2). Figure S4. DR4, TRADD and Fas knockdown confirmations (related to Fig. 1 and Fig. S2). Figure S5. Tg-induced cell death does not require TRAIL. Figure S6. DR5 and caspase-8 are strongly required for Tg-induced cell in both LNCaP and HCT116 cells (related to Fig. 1 and Fig. S2). [file 12964_2019_499_MOESM2_ESM.pdf]

Additional file 1 :

Figure S1. Chemical structures of drugs used in the current study. Chemical structures of: **(a)** Tg and the Tg analogs 8-O-debutanoyl-8-O-N-L-Leucinoyl-12-aminododecanoylthapsigargin (Leu-8ADT) and 8-O-debutanoyl-8-O-N-L-b-aspartoyl-12-aminododecanoylthapsigargin (βAsp-8ADT), **(b)** The PERK inhibitor GSK2606414, and **(c)** the JNK inhibitor JNK-IN-8.

Figure S2. Tg-induced cell death depends on DR5 and caspase-8, but not DR4, Fas, FADD, or TRADD in HCT116 cells. (**a**) HCT116 cells were transfected for 2 d with the indicated siRNAs (siCtrl = non-targeting control siRNA, siCasp8 = siCaspase-8). Subsequently, cells were treated with 100 nM Thapsigargin (Tg) or 0.01% DMSO vehicle control ("DMSO", also transfected with siCtrl) in the additional presence of 2.5 μg/ml propidium iodide to stain dead cells. Cell death was monitored and quantified with the IncuCyte ZOOM as described in Materials and Methods, and displayed as relative values normalized to those obtained in the siCtrl+Tg condition after 39 h of treatment (mean value set to 1). (**b**) HCT116 cells were transfected and treated as in (a). After 30 h of treatment with Tg or DMSO (also transfected with siCtrl), whole cell lysates were prepared and subjected to western blotting with the indicated antibodies; p55/53 Casp8 = procaspase-8, p43/41 Casp8 = cleaved caspase-8, p18 Casp8 = fully processed caspase-8, Casp3 = caspase-3 (only cleaved caspase-3 bands are shown), cl-PARP = cleaved PARP. The positions of molecular weight markers are indicated to the left, and nonspecific bands marked with an asterisk. One representative blot out of 3 independent experiments. (**c-e**) Quantifications of cleaved Caspase-8 (c), cleaved Caspase-3 (d), and cleaved PARP (e) levels, normalized to the tubulin loading control and then to the Tg+siCtrl condition (mean value set to 1). For (a, c, d, and e): Mean ± SEM from 4 (a) or 3 (c-e) independent experiments. Dots represent individual values, with a separate color for each experiment. *P < 0.05, **P < 0.01, *** P <0.001, ns; not significant, One-way ANOVA compared to the Tg+siCtrl condition.

Figure S3. Quantification of DR5 protein levels (related to Figure 1 and Figure S2). (**a** and **b**) DR5 protein levels from the experiments described in Figure 1B (a; LNCaP) and Figure S2B (b; HCT116) were quantified, normalized to the tubulin loading control, and then to the Tg+siCtrl condition (mean value set to 1). Mean ± SEM from 4 (a) or 3 (b) independent experiments. Dots represent individual values, with a separate color for each experiment. ***P < 0.001, ns; not significant, One-way ANOVA compared to the Tg+siCtrl condition.

Figure S4. DR4, TRADD and Fas knockdown confirmations (related to Figure 1 and Figure S2). (**a-f**) LNCaP (a, c, and e) or HCT116 (b, d, and f) cells were transfected for 2 d with the indicated siRNAs (siCtrl = non-targeting control siRNA). Subsequently, cells were treated with 100 nM Tg or 0.01% DMSO (also transfected with siCtrl) for 30 h, and subjected to real-time RT-PCR to quantify DR4 (a and b), TRADD (c and d), and Fas (e and f) mRNA levels. Relative mRNA levels are shown normalized to the siCtrl+DMSO condition (set to 1), i.e. the conditions shown are all with Tg treatment. Mean ± SD of triplicate measurements.

Figure S5. Tg-induced cell death does not require TRAIL. (**a**) LNCaP cells were transfected for 2 d with the indicated siRNAs (siCtrl = non-targeting control siRNA), employing two different siRNA oligoes towards TRAIL (designated by siTRAIL-1 and siTRAIL-2). Subsequently, cells were treated with 100 nM Thapsigargin (Tg) or 0.01% DMSO vehicle control ("DMSO", also transfected with siCtrl) in the additional presence of 2.5 µg/ml propidium iodide to stain dead cells. Cell death was monitored and quantified with the IncuCyte ZOOM as described in Materials and Methods, and displayed as relative values normalized to those obtained in the siCtrl+Tg condition after 48 h of treatment (mean value set to 1). Mean ± SD from 6 pictures taken from 2 biological replicates. (**b**) LNCaP cells were transfected and treated as in (a). After 30 h of treatment with Tg or DMSO (also transfected with siCtrl), TRAIL mRNA levels were determined by real-time RT-PCR as described in Materials and Methods. Relative mRNA levels are shown normalized to the DMSO control-treated condition (set to 1), i.e. the conditions shown are all with Tg treatment. Mean ± SD of triplicate measurements from one experiment.

Figure S6. DR5 and caspase-8 are strongly required for Tg-induced cell in both LNCaP and HCT116 cells (related to Figure 1 and Figure S2). (**a** and **b**) LNCaP (a) or HCT116 (b) cells were transfected for 2 d with the indicated siRNAs (siCtrl = non-targeting control siRNA), employing two different siRNA oligoes for each target (designated by -1 and -2; siCasp8-1 and siDR5-1 are the ones used in Figure 1 and Figure S2). Subsequently, cells were treated with 100 nM Thapsigargin (Tg) or 0.01% DMSO vehicle control ("DMSO", also transfected with siCtrl) in the additional presence of 2.5 µg/ml propidium iodide to stain dead cells. Cell death was monitored and quantified with the IncuCyte ZOOM as described in Materials and Methods, and displayed as relative values normalized to those obtained in the siCtrl+Tg condition after 48 h (a) or 39 h (b) of treatment (mean value set to 1). Mean ± SD from 6 pictures taken from 2 biological replicates. (**c** and **d**) LNCaP (c) or HCT116 (d) cells were transfected as in (a and b). After 30 h of treatment with Tg or DMSO (also transfected with siCtrl), whole cell lysates were prepared and subjected to western blotting with the indicated antibodies; p55/53 casp8 = procaspase-8, cl-PARP = cleaved PARP. The asterisks indicate nonspecific bands. Of note, we could in general not detect cleaved caspase-8 in LNCaP cells (in spite of testing several different antibodies), and even in HCT116 cells, the immunoblotting did not always detect cleaved caspase-8 bands, in particular after having to change to a new batch of anti-caspase-8 antibody. However, one can in this experiment appreciate the increased levels of procaspase-8 in Tg-treated DR5-silenced cells, which indicates inhibition of procaspase-8 cleavage in DR5-depleted cells. Moreover, the quantifications of successful immunoblots in HCT116 cells shown in Figure S2C clearly demonstrate that Tg-induced caspase-8 cleavage requires DR5.

Figure S1

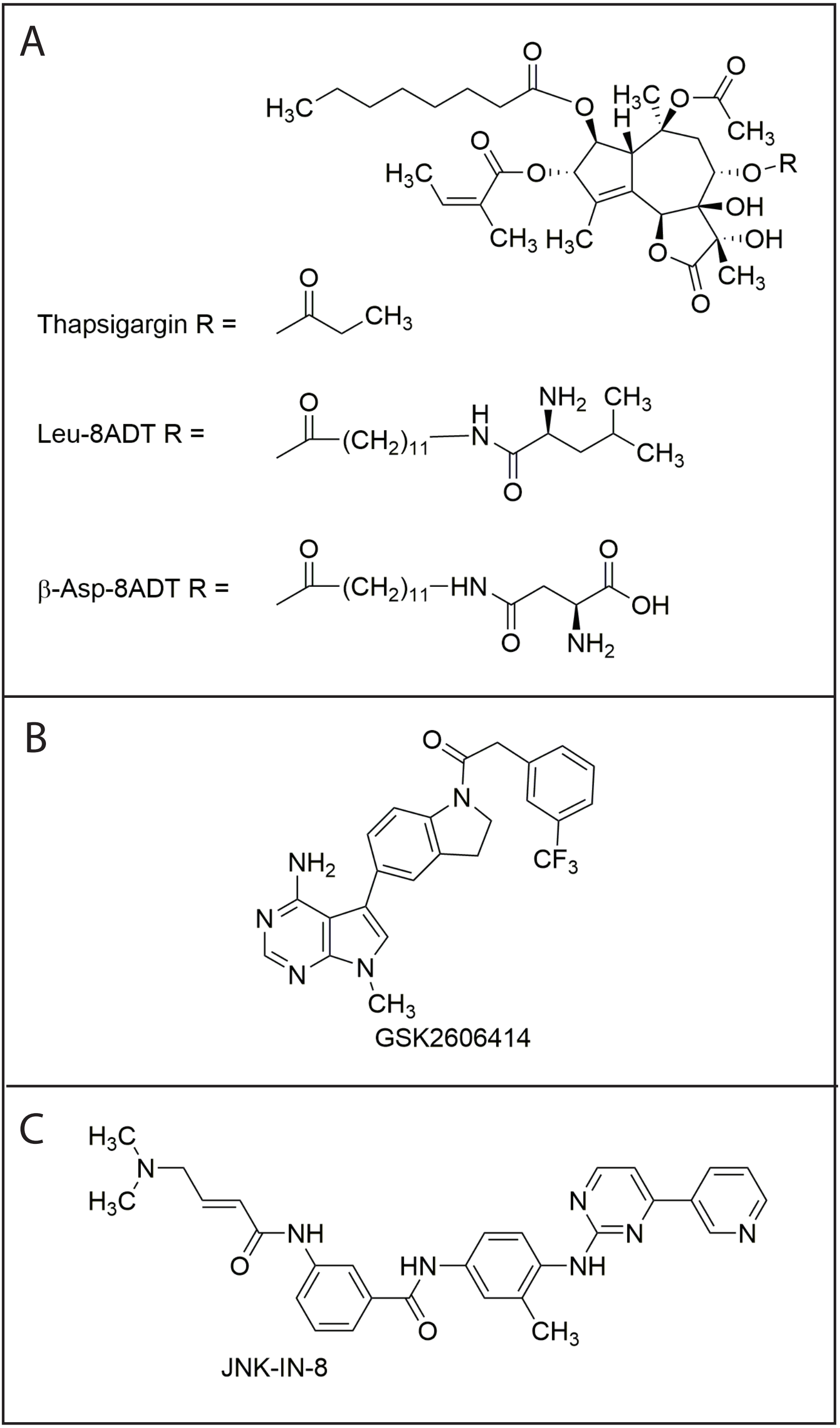

Figure S2

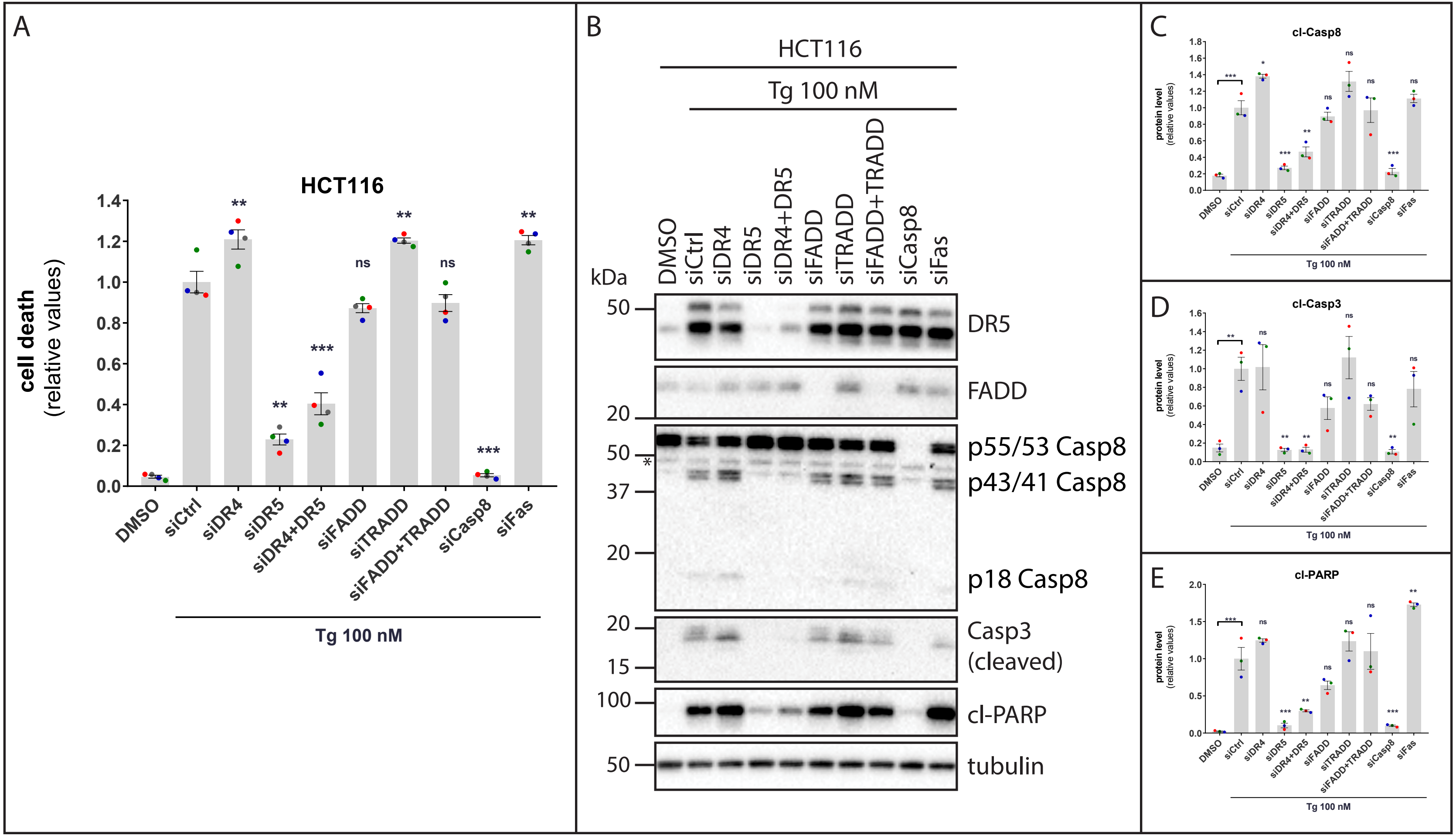

A
HCT116
cell death
(relative values)
1.4
1.2
1.0
0.8
0.6
0.4
0.2
0.0
**
**
***
ns
**
ns
***
**
DMSO
siCtrl
siDR4
siDR5
siDR4+DR5
siFADD
siTRADD
siFADD+TRADD
siCasp8
siFas
Tg 100 nM
B
HCT116
Tg 100 nM
kDa
DMSO
siCtrl
siDR4
siDR5
siDR4+DR5
siFADD
siTRADD
siFADD+TRADD
siCasp8
siFas
50
DR5
20
FADD
50
*
37
20
p55/53 Casp8
p43/41 Casp8
p18 Casp8
20
15
Casp3
(cleaved)
100
cl-PARP
50
tubulin
C
cl-Casp8
protein level
(relative values)
Tg 100 nM
D
cl-Casp3
E
cl-PARP

Figure S3

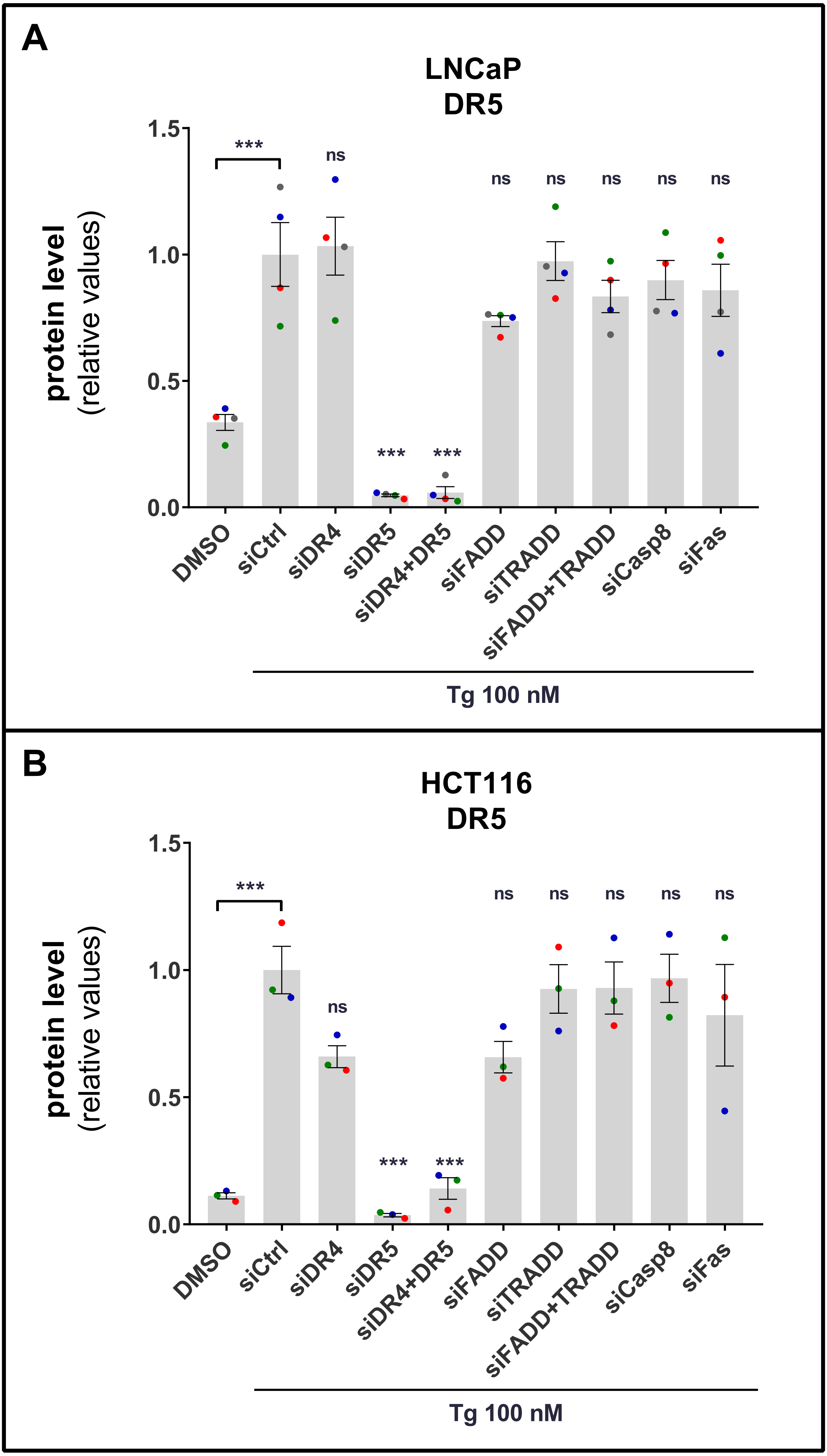

A
LNCaP
DR5
protein level
(relative values)
1.5
1.0
0.5
0.0
***
ns
***
***
ns
ns
ns
ns
ns
DMSO
siCtrl
siDR4
siDR5
siDR4+DR5
siFADD
siTRADD
siFADD+TRADD
siCasp8
siFas
Tg 100 nM
B
HCT116
DR5
protein level
(relative values)
1.5
1.0
0.5
0.0
***
ns
***
***
ns
ns
ns
ns
ns
DMSO
siCtrl
siDR4
siDR5
siDR4+DR5
siFADD
siTRADD
siFADD+TRADD
siCasp8
siFas
Tg 100 nM

Figure S4

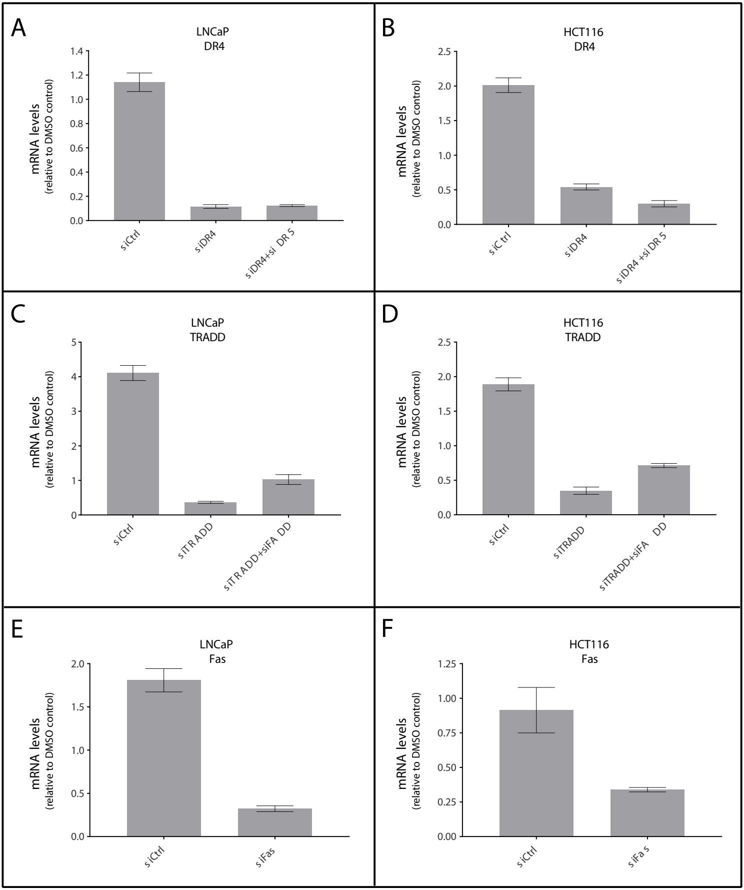
A
LNCaP
DR4
mRNA levels
(relative to DMSO control)
1.4
1.2
1.0
0.8
0.6
0.4
0.2
0.0
siCtrl
siDR4
siDR4+siDR5
B
HCT116
DR4
mRNA levels
(relative to DMSO control)
2.5
2.0
1.5
1.0
0.5
0.0
siCtrl
siDR4
siDR4+siDR5
C
LNCaP
TRADD
mRNA levels
(relative to DMSO control)
5
4
3
2
1
0
siCtrl
siTRADD
siTRADD+siFADD
D
HCT116
TRADD
mRNA levels
(relative to DMSO control)
2.5
2.0
1.5
1.0
0.5
0.0
siCtrl
siTRADD
siTRADD+siFADD
E
LNCaP
Fas
mRNA levels
(relative to DMSO control)
2.0
1.5
1.0
0.5
0.0
siCtrl
siFas
F
HCT116
Fas
mRNA levels
(relative to DMSO control)
1.25
1.00
0.75
0.50
0.25
0.00
siCtrl
siFas

Figure S5

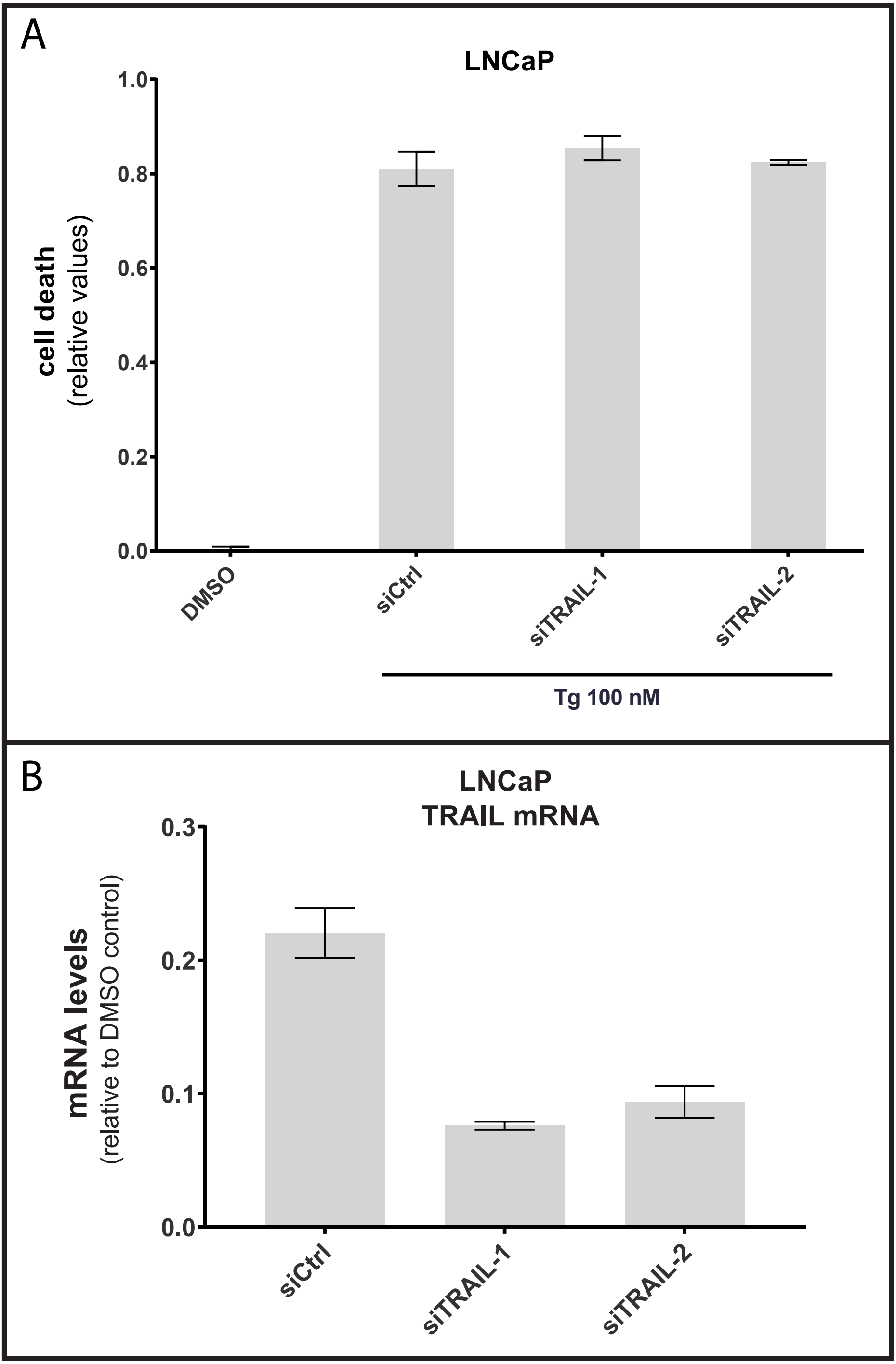
A
LNCaP
cell death
(relative values)
1.0
0.8
0.6
0.4
0.2
0.0
DMSO
siCtrl
siTRAIL-1
siTRAIL-2
Tg 100 nM
B
LNCaP
TRAIL mRNA
mRNA levels
(relative to DMSO control)
0.3
0.2
0.1
0.0
siCtrl
siTRAIL-1
siTRAIL-2

Figure S6

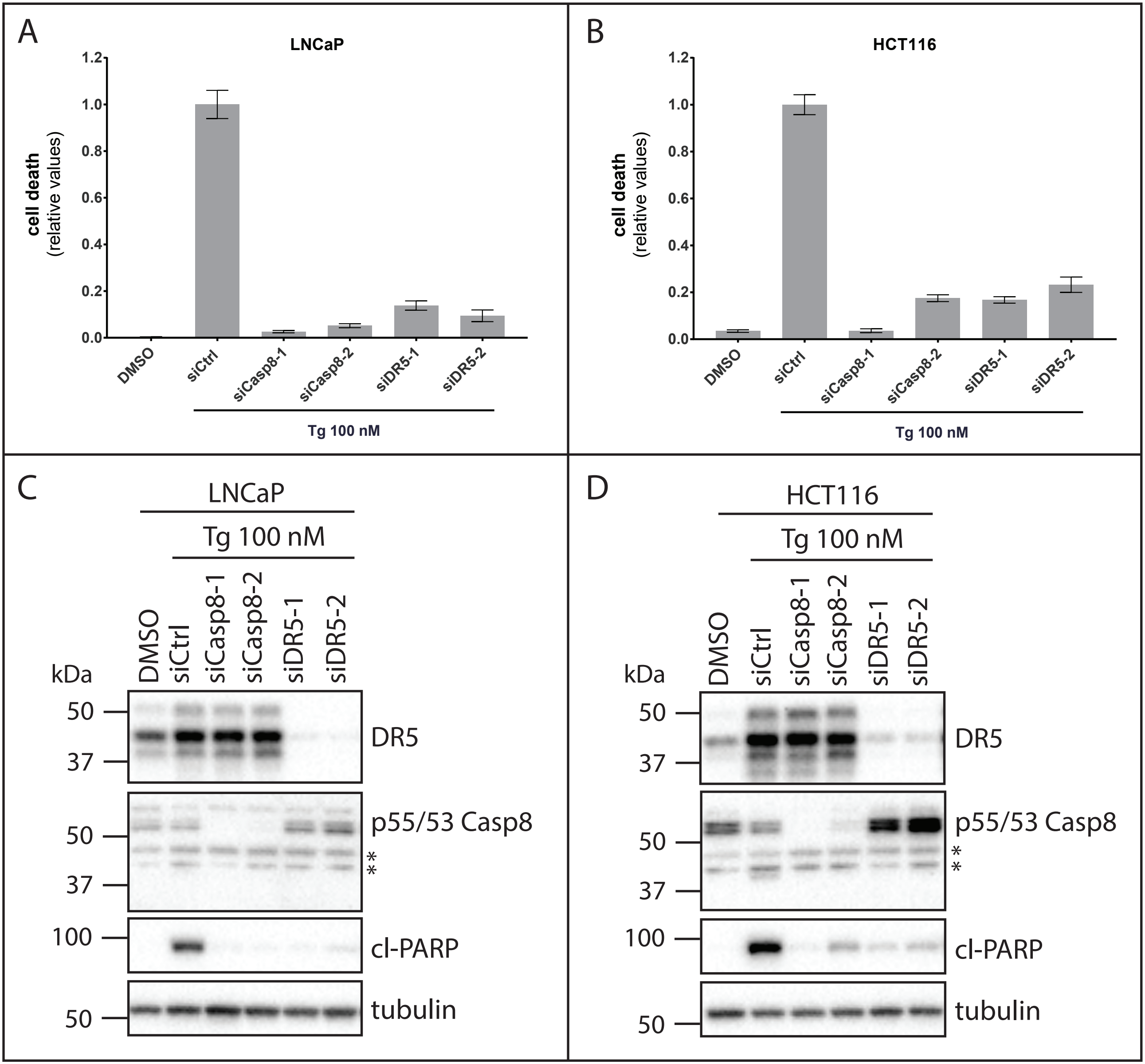

A
LNCaP
cell death (relative values)
1.2
1.0
0.8
0.6
0.4
0.2
0.0
DMSO
siCtrl
siCasp8-1
siCasp8-2
siDR5-1
siDR5-2
Tg 100 nM
B
HCT116
cell death (relative values)
DMSO
siCtrl
siCasp8-1
siCasp8-2
siDR5-1
siDR5-2
Tg 100 nM
C
LNCaP
Tg 100 nM
kDa
DMSO
siCtrl
siCasp8-1
siCasp8-2
siDR5-1
siDR5-2
50
37
DR5
p55/53 Casp8
50
37
*
*
100
cl-PARP
50
tubulin
D
HCT116
Tg 100 nM
kDa
DMSO
siCtrl
siCasp8-1
siCasp8-2
siDR5-1
siDR5-2
50
37
DR5
p55/53 Casp8
50
37
*
*
100
cl-PARP
50
tubulin
