# Supplementary material for: Cell death induced by the ER stressor thapsigargin involves death receptor 5, a non-autophagic function of MAP1LC3B, and distinct contributions from unfolded protein response components
Source: Cell Commun Signal. 2020 Jan 27;18:12. doi: 10.1186/s12964-019-0499-z (PMC6986015; doi:10.1186/s12964-019-0499-z)
Supplement: Supplementary file 3 — Additional file 2: Figure S7. LC3B is partially required for Tg-induced cell death, and LC3B depletion does not increase DR5 protein levels. Figure S8. Tg-induced cell death does not require the key autophagic membrane components ATG5, FIP200, or GABARAPs. Figure S9. Tg-induced cell death depends on PERK, ATF4 and CHOP in HCT116 cells. [file 12964_2019_499_MOESM3_ESM.pdf]

Additional file 2 :

Figure S7. LC3B is partially required for Tg-induced cell death, and LC3B depletion does not increase DR5 protein levels. (**a** and **b**) LNCaP (a) or HCT116 (b) cells were transfected for 2 d with the indicated siRNAs (siCtrl = non-targeting control siRNA, siLC3B = siLC3B-1). Subsequently, cells were treated with 100 nM Thapsigargin (Tg) or 0.01% DMSO vehicle control (“DMSO”, also transfected with siCtrl) in the additional presence of 2.5 µg/ml propidium iodide to stain dead cells. Cell death was monitored and quantified with the IncuCyte ZOOM as described in Materials and Methods, and displayed as relative values normalized to those obtained in the siCtrl+Tg condition after 48 h (a) or 39 h (b) of treatment (mean value set to 1). Mean ± SEM of 6 (a) and 3 (b) independent experiments. (**c** and **d**) LNCaP (c) or HCT116 (d) cells were transfected for 2 d with the indicated siRNAs (siCtrl = non-targeting control siRNA), employing two different siRNA oligoes for each target (designated by -1 and -2; siLC3B-2 is the oligo used in Figure 2 and siDR5-1 is the one used in Figure 1 and Figure S2). After 30 h of treatment with 100 nM Tg or 0.01% DMSO (also transfected with siCtrl), whole cell lysates were prepared and subjected to western blotting with the indicated antibodies. (**e** and **f**) Quantification of DR5 protein levels from western blots as in (c and d), normalized to the tubulin loading control and then to the Tg+siCtrl condition (mean value set to 1). The siLC3B oligo shown is siLC3B-2. Mean ± SEM from 3 independent experiments. In accordance with the reported ability of Tg to block autophagic activity, LC3B depletion did not lead to increased levels of DR5 protein, which would have been expected if DR5 was subject to LC3B-dependent autophagic degradation. For (a, b, e and f): Dots represent individual values, with a separate color for each experiment. **$P < 0.01$, ***$P < 0.001$, ns; not significant, One-way ANOVA compared to the Tg+siCtrl condition.

Figure S8. Tg-induced cell death does not require the key autophagic membrane components ATG5, FIP200, or GABARAPs. (**a**) LNCaP cells were transfected for 2 d with the indicated siRNAs (siCtrl = non-targeting control siRNA, siGABARAPs = siGABARAP + siGABARAPL1 + siGABARAPL2). Subsequently, cells were treated with 100 nM Thapsigargin (Tg) or 0.01% DMSO vehicle control (“DMSO”, also transfected with siCtrl) in the additional presence of 2.5 µg/ml propidium iodide to stain dead cells. Cell death was monitored and quantified with the IncuCyte ZOOM as described in Materials and Methods, and displayed as relative values normalized to those obtained in the siCtrl+Tg condition after 48 h of treatment (mean value set to 1). (**b** and **c**) LNCaP cells were transfected as in (a). After 30 h of treatment with 100 nM Tg or 0.01% DMSO (also transfected with siCtrl), whole cell lysates were prepared and subjected to western blotting with the indicated antibodies to confirm the knockdowns (b) and to assess DR5 protein levels (c). (**d**) HCT116 cells were transfected and treated as in (a), followed by quantification of cell death by propidium iodide staining after 39 h of treatment. (**e** and **f**) HCT116 cells were transfected as in (d). After 30 h of treatment with Tg or DMSO (also transfected with siCtrl), whole cell lysates were prepared and subjected to western blotting with the indicated antibodies to confirm the knockdowns (e) and to assess DR5 protein levels (f). For (a and d): Mean ± absolute deviation from 2 independent experiments. Dots represent individual values, with a separate color for each experiment. For (b, c, e and f): The blots are representative of 2 independent experiments. The positions of molecular weight

markers are indicated to the left of the membranes, and nonspecific bands are marked with asterisks. The membranes shown in (c and f) were cut as indicated.

Figure S9. Tg-induced cell death depends on PERK, ATF4 and CHOP in HCT116 cells. HCT116 cells were transfected for 2 d with the indicated siRNAs (siCtrl = non-targeting control siRNA), employing two different siRNA oligoes for each target (designated by -1 and -2). Subsequently, cells were treated with 0.02% DMSO (“DMSO”, also transfected with siCtrl) or 100 nM Tg in the absence of presence of 100 nM PERK inhibitor GSK2606414 (PERKi, also transfected with siCtrl), and the additional presence of 2.5 µg/ml propidium iodide in all conditions to stain dead cells. Cell death was monitored and quantified with the IncuCyte ZOOM as described in Materials and Methods, and displayed as relative values normalized to those obtained in the siCtrl+Tg condition after 39 h of treatment (mean value set to 1). Mean ± SEM from 4 independent experiments. Dots represent individual values, with a separate color for each experiment. ***$P < 0.001$, One-way ANOVA compared to the Tg+siCtrl condition.

Figure S7

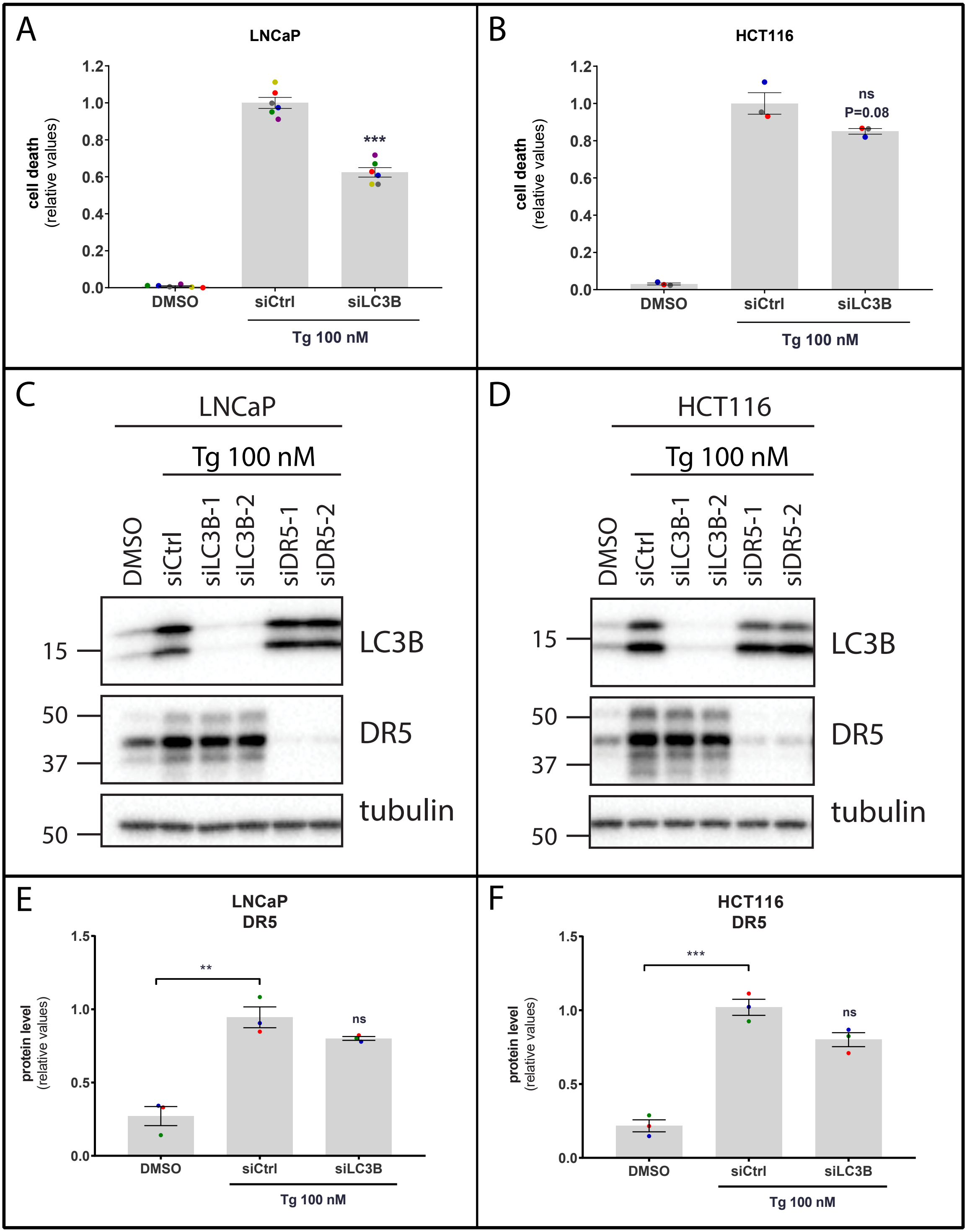
A
LNCaP
cell death
(relative values)
1.2
1.0
0.8
0.6
0.4
0.2
0.0
***
DMSO
siCtrl
siLC3B
Tg 100 nM
B
HCT116
cell death
(relative values)
1.2
1.0
0.8
0.6
0.4
0.2
0.0
ns
P=0.08
DMSO
siCtrl
siLC3B
Tg 100 nM
C
LNCaP
Tg 100 nM
DMSO
siCtrl
siLC3B-1
siLC3B-2
siDR5-1
siDR5-2
15
LC3B
50
37
DR5
50
tubulin
D
HCT116
Tg 100 nM
DMSO
siCtrl
siLC3B-1
siLC3B-2
siDR5-1
siDR5-2
15
LC3B
50
37
DR5
50
tubulin
E
LNCaP
DR5
protein level
(relative values)
1.5
1.0
0.5
0.0
**
ns
DMSO
siCtrl
siLC3B
Tg 100 nM
F
HCT116
DR5
protein level
(relative values)
1.5
1.0
0.5
0.0
***
ns
DMSO
siCtrl
siLC3B
Tg 100 nM

Figure S8

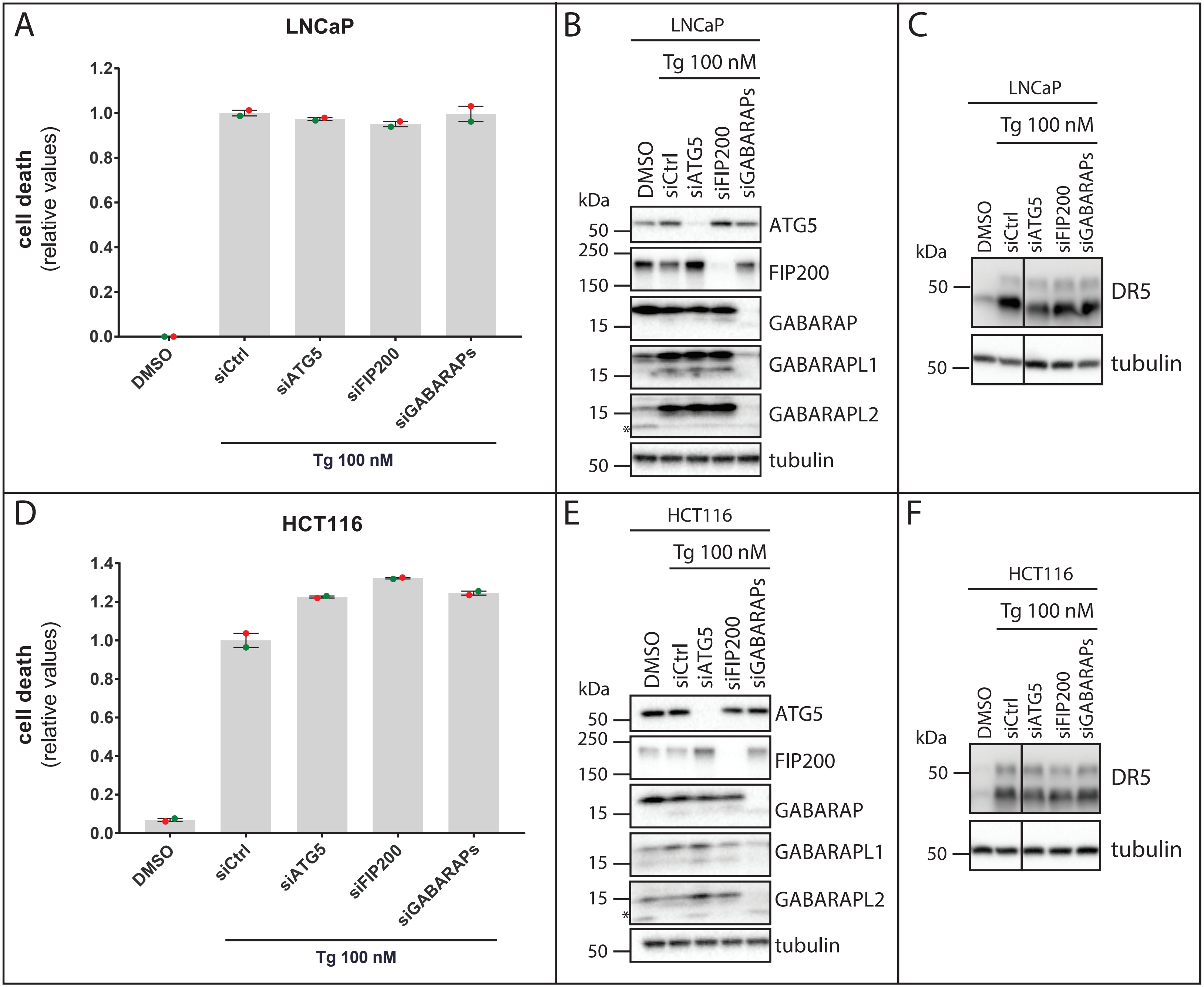

A
LNCaP
cell death (relative values)
1.2
1.0
0.8
0.6
0.4
0.2
0.0
DMSO
siCtrl
siATG5
siFIP200
siGABARAPs
Tg 100 nM
B
LNCaP
Tg 100 nM
DMSO
siCtrl
siATG5
siFIP200
siGABARAPs
kDa
50
250
150
15
15
15
*
50
ATG5
FIP200
GABARAP
GABARAPL1
GABARAPL2
tubulin
C
LNCaP
Tg 100 nM
DMSO
siCtrl
siATG5
siFIP200
siGABARAPs
kDa
50
50
DR5
tubulin
D
HCT116
cell death (relative values)
1.4
1.2
1.0
0.8
0.6
0.4
0.2
0.0
DMSO
siCtrl
siATG5
siFIP200
siGABARAPs
Tg 100 nM
E
HCT116
Tg 100 nM
DMSO
siCtrl
siATG5
siFIP200
siGABARAPs
kDa
50
250
150
15
15
15
*
50
ATG5
FIP200
GABARAP
GABARAPL1
GABARAPL2
tubulin
F
HCT116
Tg 100 nM
DMSO
siCtrl
siATG5
siFIP200
siGABARAPs
kDa
50
50
DR5
tubulin

Figure S9

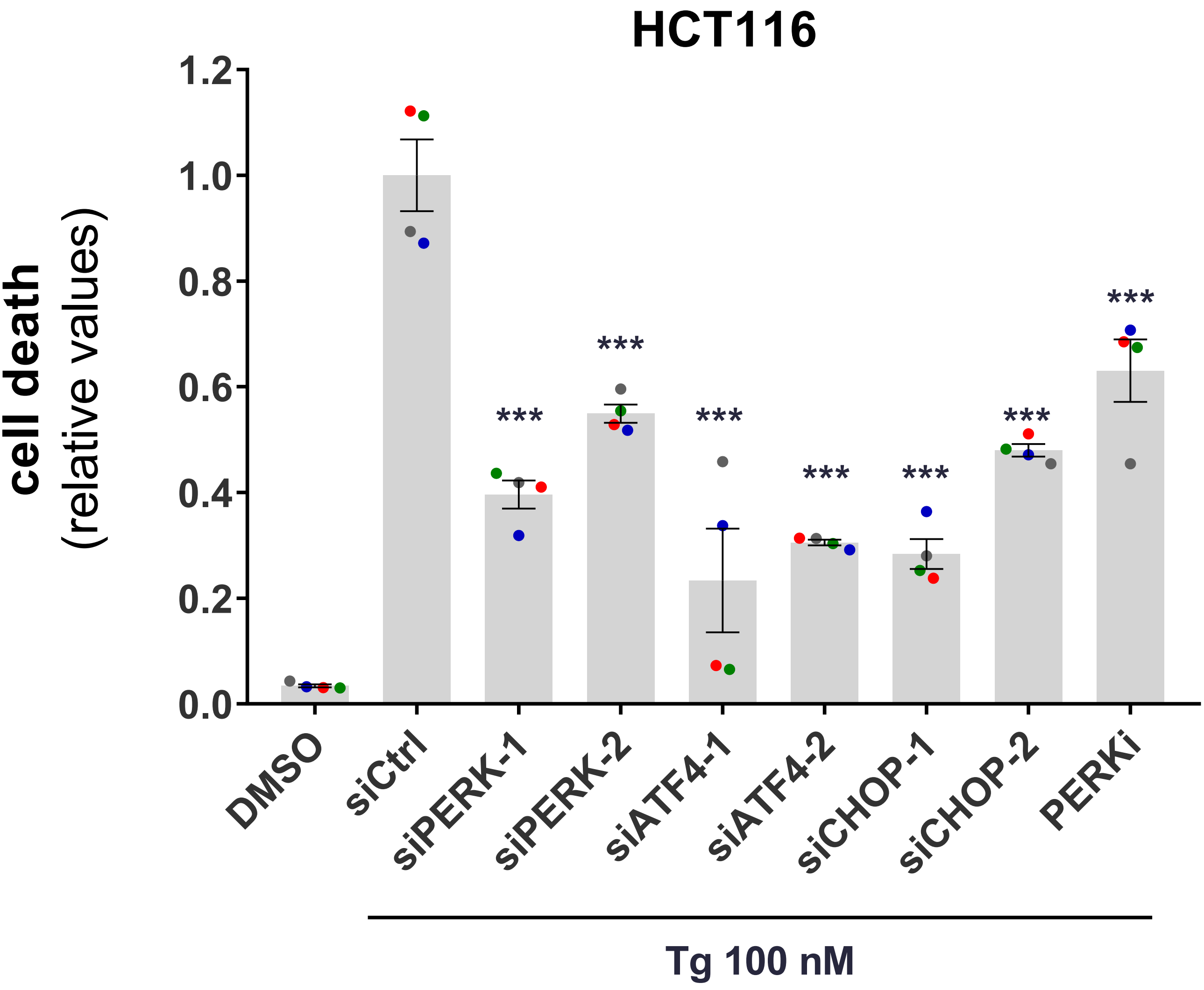
HCT116
cell death
(relative values)
1.2
1.0
0.8
0.6
0.4
0.2
0.0
***
DMSO
siCtrl
siPERK-1
siPERK-2
siATF4-1
siATF4-2
siCHOP-1
siCHOP-2
PERKi
Tg 100 nM
