# Supplementary material for: Cell death induced by the ER stressor thapsigargin involves death receptor 5, a non-autophagic function of MAP1LC3B, and distinct contributions from unfolded protein response components
Source: Cell Commun Signal. 2020 Jan 27;18:12. doi: 10.1186/s12964-019-0499-z (PMC6986015; doi:10.1186/s12964-019-0499-z)
Supplement: Supplementary file 4 — Additional file 3: Figure S10. Tg-induced caspase activation in HCT116 cells requires PERK, ATF4 and CHOP, whereas Tg-mediated upregulation DR5- and LC3B protein levels depends on individual contributions from ATF4 and CHOP, but not PERK. [file 12964_2019_499_MOESM4_ESM.pdf]

Additional file 3 :

Figure S10. Tg-induced caspase activation in HCT116 cells requires PERK, ATF4 and CHOP, whereas Tg-mediated upregulation DR5 and LC3B protein levels depends on individual contributions from ATF4 and CHOP, but not PERK. (**a**) HCT116 cells were transfected for 2 d with the indicated siRNAs (siCtrl = non-targeting control siRNA), employing two different siRNA oligoes for each target (designated by -1 and -2). After 30 h of treatment with 100 nM Tg or 0.02% DMSO vehicle control (also transfected with siCtrl), whole cell lysates were prepared and subjected to western blotting with the indicated antibodies; Casp3 = caspase-3 (only cleaved caspase-3 bands are shown), cl-PARP = cleaved PARP. The positions of molecular weight markers are indicated to the left. The shift towards the slower migrating PERK band in Tg-treated cells reflects induction of PERK phosphorylation by Tg. One representative blot out of at least 3 independent experiments. (**b-g**) Quantifications of western blots from (a), normalized to the tubulin loading control and then to the siCtrl+Tg condition. Mean ± SEM from 4 (b-d, f, and g) or 3 (e) independent experiments. Dots represent individual values, with a separate color for each experiment. **P* < 0.05, ***P* < 0.01, ****P* < 0.001, ns; not significant, One-way ANOVA compared to the Tg+siCtrl condition.

Figure S10

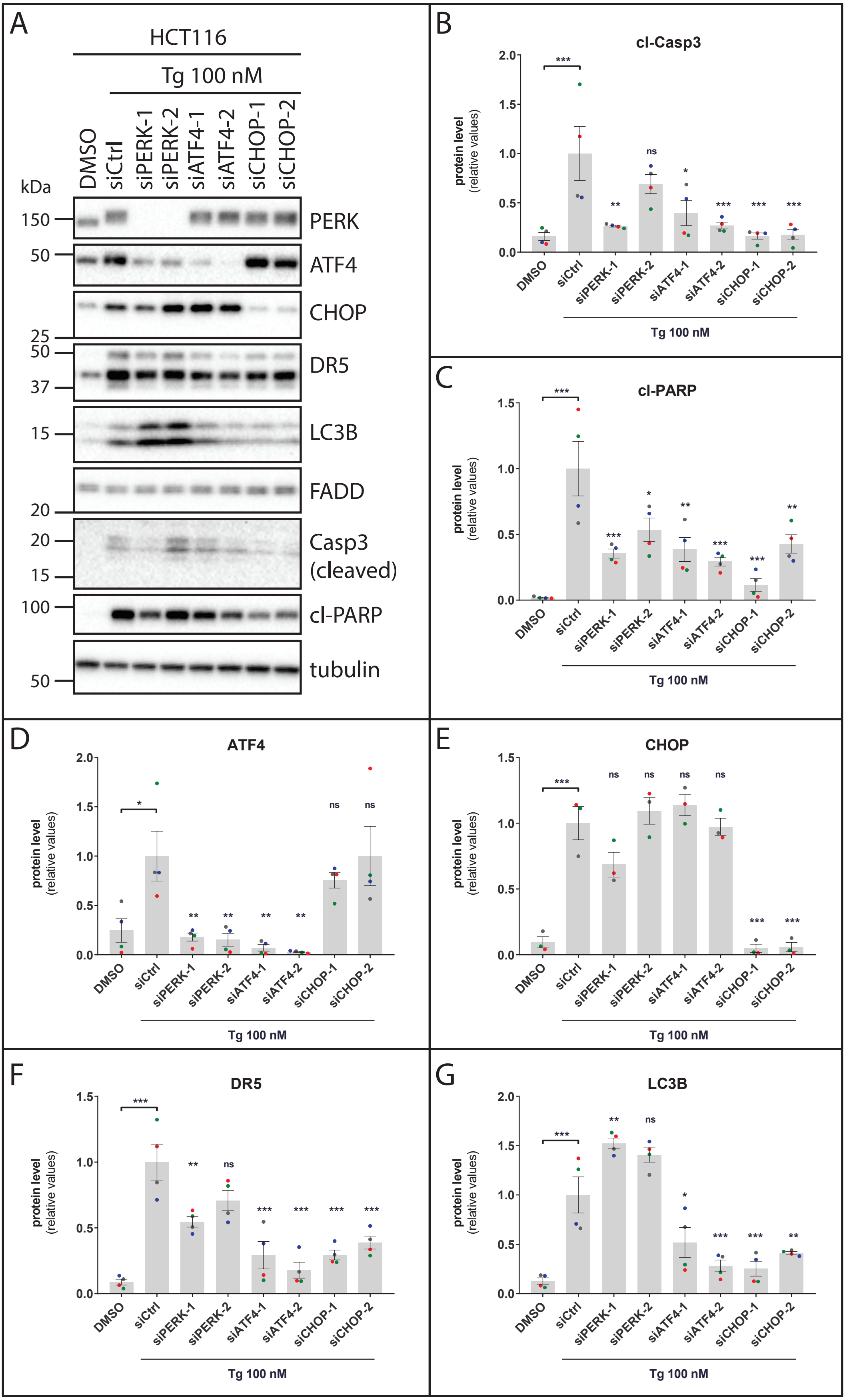
A
HCT116
Tg 100 nM
DMSO
siCtrl
siPERK-1
siPERK-2
siATF4-1
siATF4-2
siCHOP-1
siCHOP-2
kDa
150
50
25
50
37
15
20
20
15
100
50
PERK
ATF4
CHOP
DR5
LC3B
FADD
Casp3 (cleaved)
cl-PARP
tubulin
B
cl-Casp3
C
cl-PARP
D
ATF4
E
CHOP
F
DR5
G
LC3B
protein level (relative values)
Tg 100 nM
ns
*
**
***
