# Supplementary material for: Cell death induced by the ER stressor thapsigargin involves death receptor 5, a non-autophagic function of MAP1LC3B, and distinct contributions from unfolded protein response components
Source: Cell Commun Signal. 2020 Jan 27;18:12. doi: 10.1186/s12964-019-0499-z (PMC6986015; doi:10.1186/s12964-019-0499-z)
Supplement: Supplementary file 5 — Additional file 4: Figure S11. Regulation of Tg-mediated upregulation of DR5- and LC3B protein and mRNA levels by PERK, ATF4 and CHOP at an early time point (6 h). [file 12964_2019_499_MOESM5_ESM.pdf]

Additional file 4 :

Figure S11. Regulation of Tg-mediated upregulation of DR5- and LC3B protein and mRNA levels by PERK, ATF4 and CHOP at an early time point (6 h). (**a-f**) LNCaP (a-c) or HCT116 (d-f) cells were transfected for 2 d with the indicated siRNAs (siCtrl = non-targeting control siRNA), employing two different siRNA oligoes for each target (designated by -1 and -2). Subsequently, cells were treated with 100 nM Tg or 0.01% DMSO (also transfected with siCtrl) for 6 h. All samples were subjected to western blotting with the indicated antibodies (a and d; the blots are representative of 2 independent experiments), whereas selected samples (siCtrl+DMSO, siCtrl+Tg, siPERK-2+Tg, siATF4-1+Tg, siCHOP-1+Tg) were subjected to real-time RT-PCR for quantification of DR5 (b and e) and LC3B (c and f) mRNA levels. Relative mRNA levels are shown normalized to the siCtrl+DMSO condition (set to 1 and indicated by the dotted line in the graphs), i.e. the conditions shown are all with Tg treatment. Mean ± SD of triplicate measurements.

Figure S11

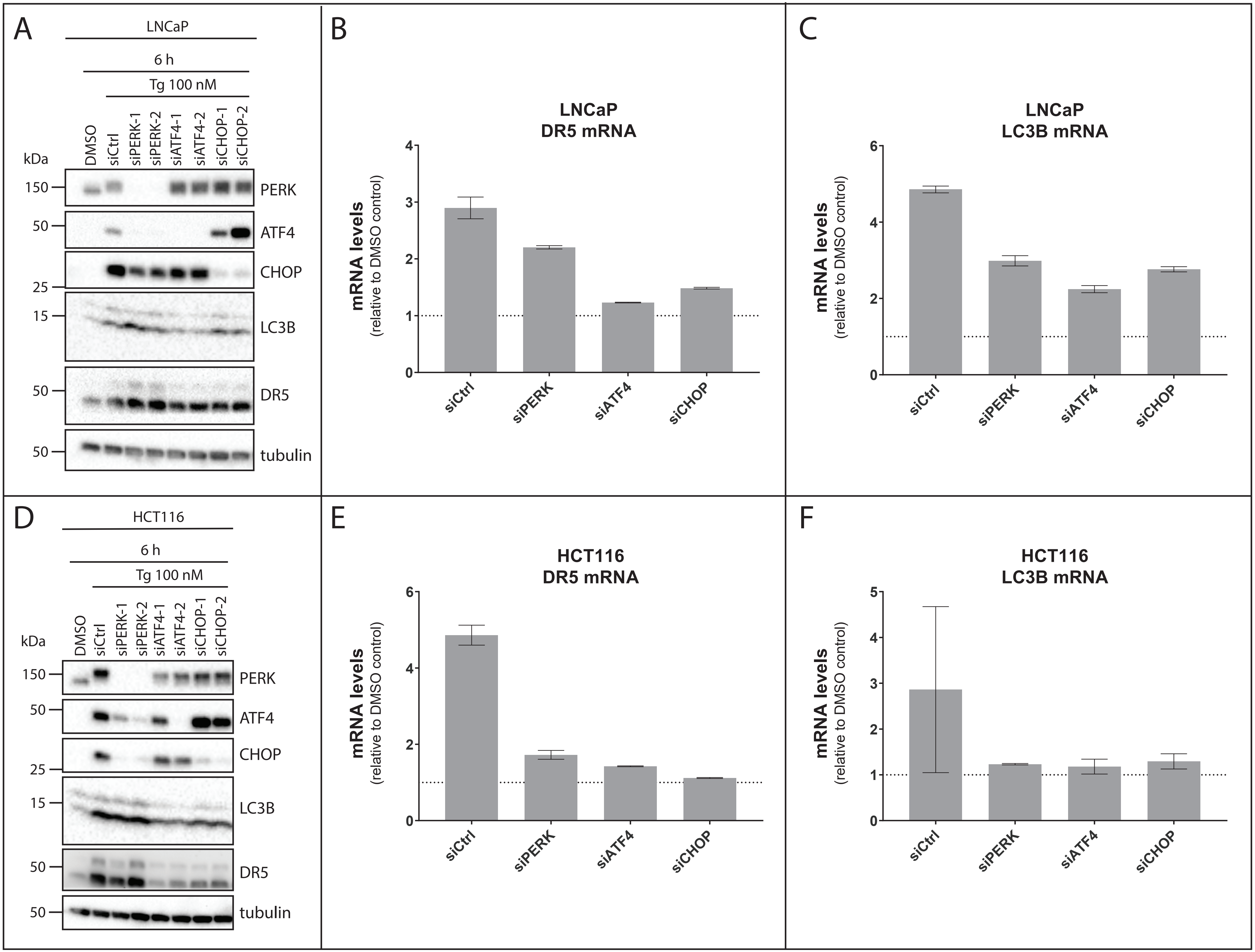

A
LNCaP
6 h
Tg 100 nM
DMSO
siCtrl
siPERK-1
siPERK-2
siATF4-1
siATF4-2
siCHOP-1
siCHOP-2
kDa
150
50
25
15
50
50
PERK
ATF4
CHOP
LC3B
DR5
tubulin
B
LNCaP
DR5 mRNA
mRNA levels
(relative to DMSO control)
0
1
2
3
4
siCtrl
siPERK
siATF4
siCHOP
C
LNCaP
LC3B mRNA
mRNA levels
(relative to DMSO control)
0
2
4
6
siCtrl
siPERK
siATF4
siCHOP
D
HCT116
6 h
Tg 100 nM
DMSO
siCtrl
siPERK-1
siPERK-2
siATF4-1
siATF4-2
siCHOP-1
siCHOP-2
kDa
150
50
25
15
50
50
PERK
ATF4
CHOP
LC3B
DR5
tubulin
E
HCT116
DR5 mRNA
mRNA levels
(relative to DMSO control)
0
2
4
6
siCtrl
siPERK
siATF4
siCHOP
F
HCT116
LC3B mRNA
mRNA levels
(relative to DMSO control)
0
1
2
3
4
5
siCtrl
siPERK
siATF4
siCHOP
