# Supplementary material for: Cell death induced by the ER stressor thapsigargin involves death receptor 5, a non-autophagic function of MAP1LC3B, and distinct contributions from unfolded protein response components
Source: Cell Commun Signal. 2020 Jan 27;18:12. doi: 10.1186/s12964-019-0499-z (PMC6986015; doi:10.1186/s12964-019-0499-z)
Supplement: Supplementary file 6 — Additional file 5: Figure S12. Tg-mediated upregulation of DR5- and LC3B mRNA levels requires PERK, ATF4 and CHOP in LNCaP and HCT116 cells. Figure S13. IRE1 and ATF6 knockdown confirmations (related to Fig. 5). Figure S14. Tg-mediated caspase activation and upregulation of DR5 and LC3B does not require IRE1, XBP1, ATF6, or JNK in HCT116 cells. Figure S15. Tg rapidly enhances XBP1s mRNA levels in an IRE1-dependent manner in LNCaP cells (related to Fig. 8). Figure S16. Cell death induced by the therapeutically relevant Tg analogs Leu-8ADT and βAsp-8ADT requires DR5 and caspase-8 in LNCaP and HCT116 cells, and partially requires FADD and Fas in LNCaP cells, whereas DR4 and TRADD are not required in any of the cell lines. Figure S17. Cell death induced by Leu-8ADT and βAsp-8ADT requires PERK, ATF4, and CHOP in LNCaP and HCT116 cells. Figure S18. Cell death induced by Leu-8ADT and βAsp-8ADT involves IRE1, XBP1, and JNK in LNCaP, but not HCT116 cells. [file 12964_2019_499_MOESM6_ESM.pdf]

# Additional file 5 :

**Figure S12. Tg-mediated upregulation of DR5- and LC3B mRNA levels requires PERK, ATF4 and CHOP in LNCaP and HCT116 cells.** (**a-d**) In parallel to the western blot experiments described in Figure 4 (LNCaP) and Figure S10 (HCT116) (for both cell lines; cells were transfected with the indicated siRNAs for 2 d, followed by treatment with 100 nM Tg or 0.02% DMSO for 30 h), the samples were subjected to real-time RT-PCR for quantification of DR5 (a and c) and LC3B (b and d) mRNA levels. Relative mRNA levels are shown normalized to the siCtrl+DMSO condition (set to 1 and indicated by the dotted line in the graphs), i.e. the conditions shown are all with Tg treatment. Mean ± SEM from 4 independent experiments. Dots represent individual values, with a separate color for each experiment. **P* < 0.05, ***P* < 0.01, ****P* < 0.001, ns; not significant, One-way ANOVA compared to the Tg+siCtrl condition.

**Figure S13. IRE1 and ATF6 knockdown confirmations (related to Figure 5).** (**a-d**) LNCaP (a and b) or HCT116 (c and d) cells were transfected for 2 d with the indicated siRNAs (siCtrl = non-targeting control siRNA), employing two different siRNA oligoes for each target (designated by -1 and -2). Subsequently, cells were treated with 100 nM Tg or 0.01% DMSO (also transfected with siCtrl) for 30 h, and subjected to real-time RT-PCR to quantify IRE1 (a and c) and ATF6 (b and d) mRNA levels. Relative mRNA levels are shown normalized to the siCtrl+DMSO condition (set to 1), i.e. the conditions shown are all with Tg treatment. Mean ± SD of triplicate measurements.

**Figure S14. Tg-mediated caspase activation and upregulation of DR5 and LC3B does not require IRE1, XBP1, ATF6, or JNK in HCT116 cells.** (**a**) HCT116 cells were transfected for 2 d with the indicated siRNAs (siCtrl = non-targeting control siRNA), employing two different siRNA oligoes for each target (designated by -1 and -2). After 30 h of treatment with 0.02% DMSO ("DMSO", also transfected with siCtrl) or 100 nM Tg in the absence of presence of 0.5 µM JNK inhibitor JNK-IN-8 (JNKi, also transfected with siCtrl), whole cell lysates were prepared and subjected to western blotting with the indicated antibodies; Casp3 = caspase-3 (only cleaved caspase-3 bands are shown), cl-PARP = cleaved PARP, p-JNK = phospho-JNK. The positions of molecular weight markers are indicated to the left. In HCT116 cells, the anti-phospho-JNK antibody detects a nonspecific band (indicated by an asterisk) whose migration partly overlaps with that of the fastest migrating, ~p46 p-JNK band (likely representing p-JNK1 isoforms). The nonspecific band migrates slightly faster than the ~p46 p-JNK band. Nevertheless, the two bands merge into one. In the top ~p54 p-JNK bands (likely representing p-JNK2 isoforms) one can easier appreciate the reduction in p-JNK levels that is obtained upon IRE1- or XBP1 (but not ATF6) depletion. In the presence of JNK-IN-8, the two p-JNK-specific bands shift towards slightly slower migration due to covalent binding of the inhibitor to the JNK1 and JNK2 isoforms. One representative blot out of at least 3 independent experiments. (**b-g**) Quantifications of western blots from (a), normalized to the tubulin loading control and then to the siCtrl+Tg condition. Mean ± SEM from 4 (b, c, d, and f) or 3 (e and g) independent experiments. Dots represent individual values, with a separate color for each experiment. **P* < 0.05, ***P* < 0.01, ****P* < 0.001, ns; not significant, One-way ANOVA compared to the Tg+siCtrl condition.

**Figure S15. Tg rapidly enhances XBP1s mRNA levels in an IRE1-dependent manner (related to Figure 8).** In parallel to the western blot experiments described in Figure 8 (where LNCaP cells were transfected with the indicated siRNAs for 2 d, followed by treatment with 100 nM Tg or 0.02% DMSO for 30 min, 6 h, or 30 h), samples were subjected to real-time RT-PCR for quantification of IRE1 (a), XBP1 (b; unspliced XBP1), and XBP1s (c; spliced XBP1) mRNA levels. Relative mRNA levels are shown normalized to the siCtrl+DMSO condition (set to 1 and indicated by the dotted line in the graphs), i.e. the conditions shown are all with Tg treatment (for either 30 min or 6 h, as indicated). Mean ± SD of triplicate measurements. Note that the Tg-induced ~5-fold increase in XBP1s mRNA levels at 30 min is efficiently abolished by IRE1 depletion (c). At later time points (6 h, 30 h), Tg strongly upregulates IRE1 mRNA and protein levels (see (a), Figure S13, and Figures 6, 7 and 8), which in spite of efficient knockdown leads to higher levels of IRE1 also in the siIRE1-transfected condition, thus likely at least in part explaining the less efficient (only partial) abolishment of Tg-mediated enhancement of XBP1s levels at the later time points.

**Figure S16. Cell death induced by the therapeutically relevant Tg analogs Leu-8ADT and βAsp-8ADT requires DR5 and caspase-8 in LNCaP and HCT116 cells, and partially requires FADD and Fas in LNCaP cells, whereas DR4 and TRADD are not required in any of the cell lines.** (**a-f**) LNCaP (a-c) or HCT116 (d-f) cells were transfected for 2 d with the indicated siRNAs (siCtrl = non-targeting control siRNA). Subsequently, cells were treated with 100 nM Tg (a and d), 500 nM Leu-8ADT (b and e), or 1 µM βAsp-8ADT (c and f), or 0.01% DMSO vehicle control ("DMSO", also transfected with siCtrl) in the additional presence of 2.5 µg/ml propidium iodide to stain dead cells. Cell death was monitored and quantified with the IncuCyte ZOOM as described in Materials and Methods, and displayed as relative values normalized to those obtained in the siCtrl+Tg condition after 48 h (a-c) or 39 h (d-f) of treatment (mean value set to 1). Mean ± SEM from 3 (b, c, e, and f) or 4 (a and d) independent experiments. Dots represent individual values, with a separate color for each experiment. *$P < 0.05$, **$P < 0.01$, ***$P < 0.001$, ns; not significant, One-way ANOVA compared to the Tg/Tg analog+siCtrl condition. Note: The results shown with Tg (a and d) are identical to those shown in Fig. 1 and Fig. S2, and are re-shown here to facilitate direct visual comparison to the results obtained with the Tg analogs.

**Figure S17. Cell death induced by Leu-8ADT and βAsp-8ADT requires PERK, ATF4, and CHOP in LNCaP and HCT116 cells.** (**a-f**) LNCaP (a-c) or HCT116 (d-f) cells were transfected for 2 d with the indicated siRNAs (siCtrl = non-targeting control siRNA), employing two different siRNA oligoes for each target (designated by -1 and -2). Subsequently, cells were treated with 0.02% DMSO ("DMSO", also transfected with siCtrl) or 100 nM Tg (a and d), 500 nM Leu-8ADT (b and e), or 1 µM βAsp-8ADT (c and f) in the absence of presence of 100 nM PERK inhibitor GSK2606414 (PERKi, also transfected with siCtrl) and the additional presence of 2.5 µg/ml propidium iodide in all conditions to stain dead cells. Cell death was monitored and quantified with the IncuCyte ZOOM as described in Materials and Methods, and displayed as relative values normalized to those obtained in the siCtrl+Tg condition after 48 h (a-c) or 39 h (d-f) of treatment (mean value set to 1). Mean ± SEM from 4 independent experiments. Dots represent individual values, with a separate color for each experiment. **$P < 0.01$, ***$P <$

0.001, One-way ANOVA compared to the Tg/Tg analog+siCtrl condition. Note: The results shown with Tg (a and d) are identical to those shown in Fig. 3 and Fig. S9, and are re-shown here to facilitate direct visual comparison to the results obtained with the Tg analogs.

**Figure S18. Cell death induced by Leu-8ADT and βAsp-8ADT involves IRE1, XBP1, and JNK in LNCaP, but not HCT116 cells.** (**a**-**f**) LNCaP (a-c) or HCT116 (d-f) cells were transfected for 2 d with the indicated siRNAs (siCtrl = non-targeting control siRNA), employing two different siRNA oligoes for each target (designated by -1 and -2). Subsequently, cells were treated with 0.02% DMSO ("DMSO", also transfected with siCtrl) or 100 nM Tg (a and d), 500 nM Leu-8ADT (b and e), or 1 µM βAsp-8ADT (c and f) in the absence of presence of 0.5 µM JNK inhibitor JNK-IN-8 (JNKi, also transfected with siCtrl) and the additional presence of 2.5 µg/ml propidium iodide in all conditions to stain dead cells. Cell death was monitored and quantified with the IncuCyte ZOOM as described in Materials and Methods, and displayed as relative values normalized to those obtained in the siCtrl+Tg condition after 48 h (a-c) or 39 h (d-f) of treatment (mean value set to 1). Mean ± SEM from 4 independent experiments. Dots represent individual values, with a separate color for each experiment. *$P < 0.05$, **$P < 0.01$, ***$P < 0.001$, ns; not significant, One-way ANOVA compared to the Tg/Tg analog+siCtrl condition. Note: The results shown with Tg (a and d) are identical to those shown in Fig. 5, and are re-shown here to facilitate direct visual comparison to the results obtained with the Tg analogs.

Figure S12

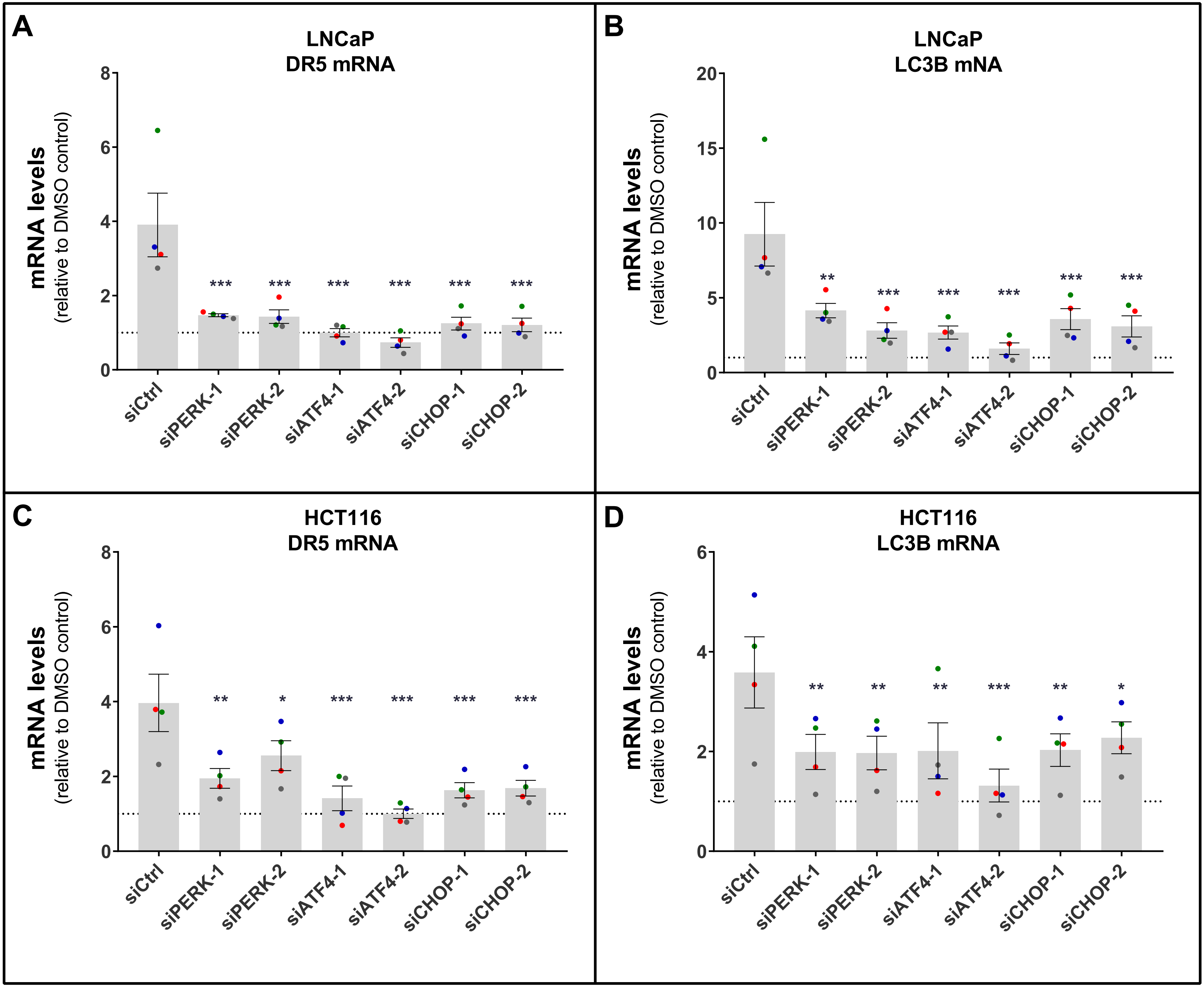
A
LNCaP
DR5 mRNA
B
LNCaP
LC3B mNA
C
HCT116
DR5 mRNA
D
HCT116
LC3B mRNA
mRNA levels
(relative to DMSO control)
siCtrl
siPERK-1
siPERK-2
siATF4-1
siATF4-2
siCHOP-1
siCHOP-2
***
**
*

Figure S13

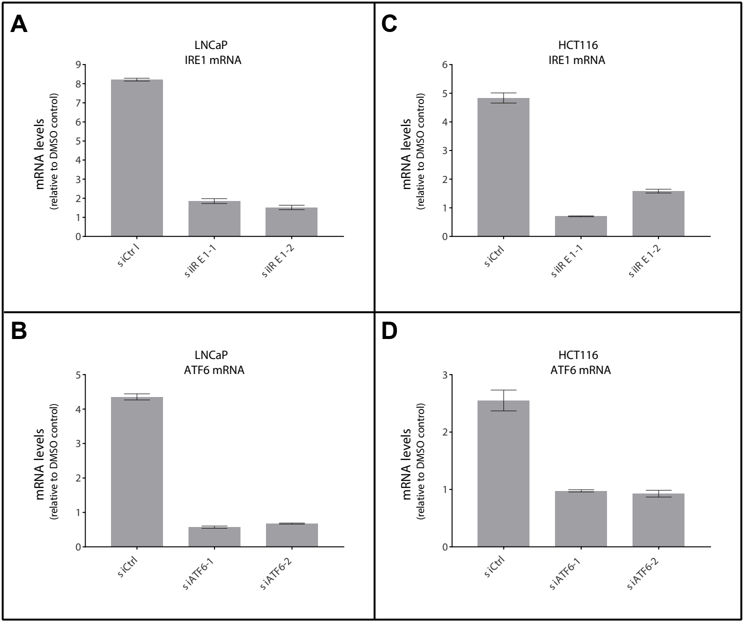

A
LNCaP
IRE1 mRNA
mRNA levels
(relative to DMSO control)
0
1
2
3
4
5
6
7
8
9
siCtrl
siIRE1-1
siIRE1-2
B
LNCaP
ATF6 mRNA
mRNA levels
(relative to DMSO control)
0
1
2
3
4
5
siCtrl
siATF6-1
siATF6-2
C
HCT116
IRE1 mRNA
mRNA levels
(relative to DMSO control)
0
1
2
3
4
5
6
siCtrl
siIRE1-1
siIRE1-2
D
HCT116
ATF6 mRNA
mRNA levels
(relative to DMSO control)
0
1
2
3
siCtrl
siATF6-1
siATF6-2

Figure S14

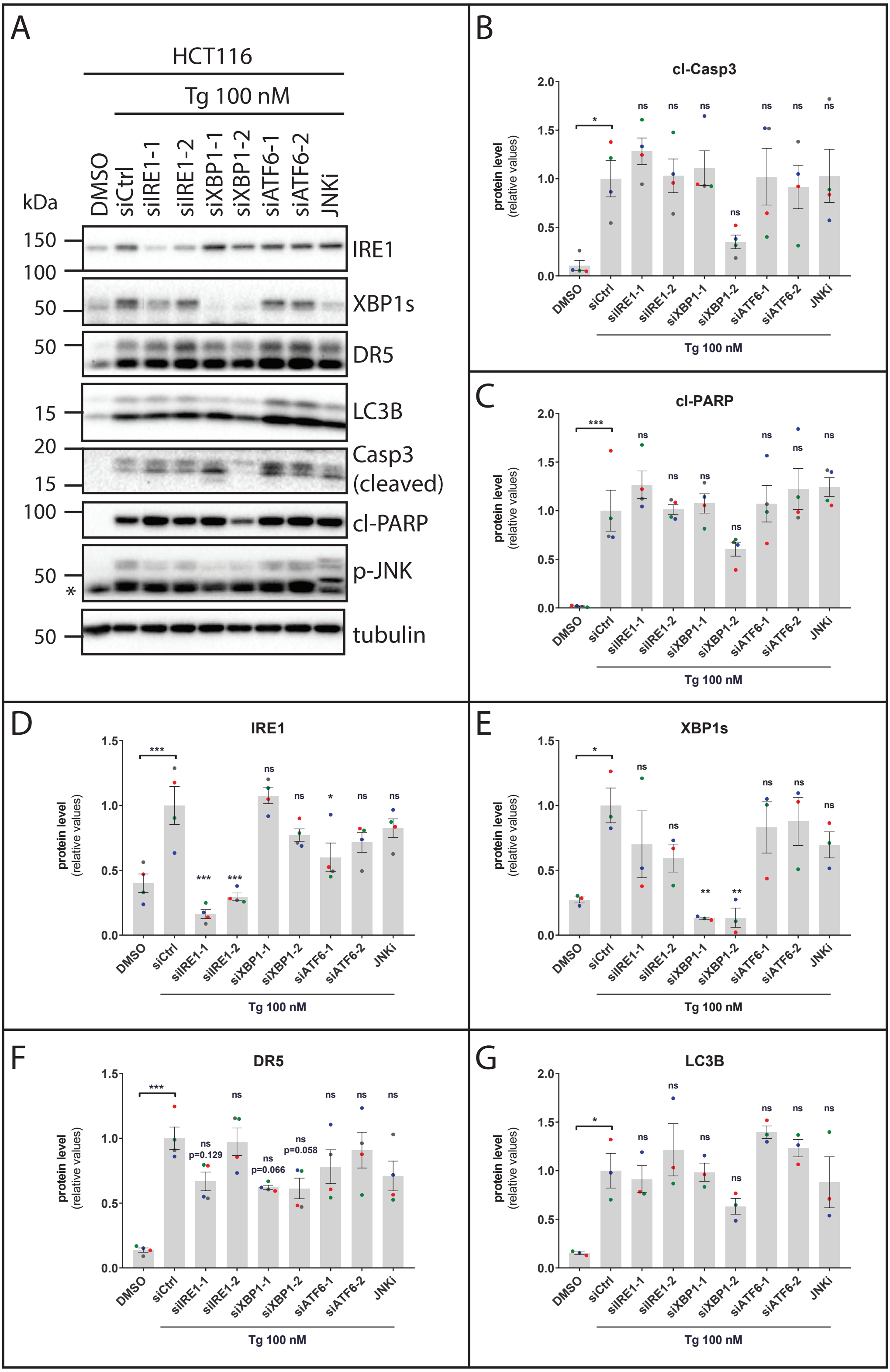
A
HCT116
Tg 100 nM
kDa
DMSO
siCtrl
siIRE1-1
siIRE1-2
siXBP1-1
siXBP1-2
siATF6-1
siATF6-2
JNKi
150
100
IRE1
50
XBP1s
50
DR5
15
LC3B
20
15
Casp3 (cleaved)
100
cl-PARP
50
*
p-JNK
50
tubulin
B
cl-Casp3
C
cl-PARP
D
IRE1
E
XBP1s
F
DR5
p=0.129
p=0.066
p=0.058
G
LC3B
protein level (relative values)
ns
*
**
***
Tg 100 nM

# Figure S15

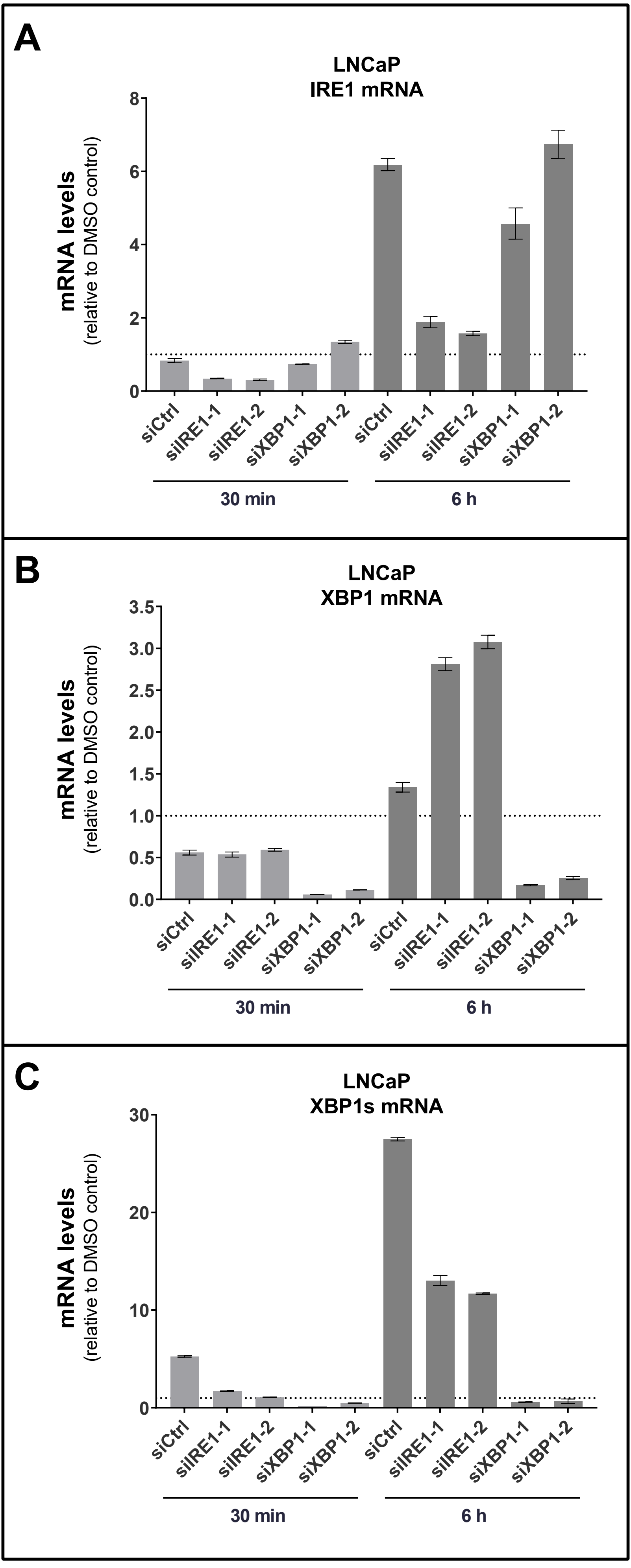
A
LNCaP
IRE1 mRNA
mRNA levels
(relative to DMSO control)
0
2
4
6
8
siCtrl
siIRE1-1
siIRE1-2
siXBP1-1
siXBP1-2
siCtrl
siIRE1-1
siIRE1-2
siXBP1-1
siXBP1-2
30 min
6 h
B
LNCaP
XBP1 mRNA
mRNA levels
(relative to DMSO control)
0.0
0.5
1.0
1.5
2.0
2.5
3.0
3.5
siCtrl
siIRE1-1
siIRE1-2
siXBP1-1
siXBP1-2
siCtrl
siIRE1-1
siIRE1-2
siXBP1-1
siXBP1-2
30 min
6 h
C
LNCaP
XBP1s mRNA
mRNA levels
(relative to DMSO control)
0
10
20
30
siCtrl
siIRE1-1
siIRE1-2
siXBP1-1
siXBP1-2
siCtrl
siIRE1-1
siIRE1-2
siXBP1-1
siXBP1-2
30 min
6 h

Figure S16

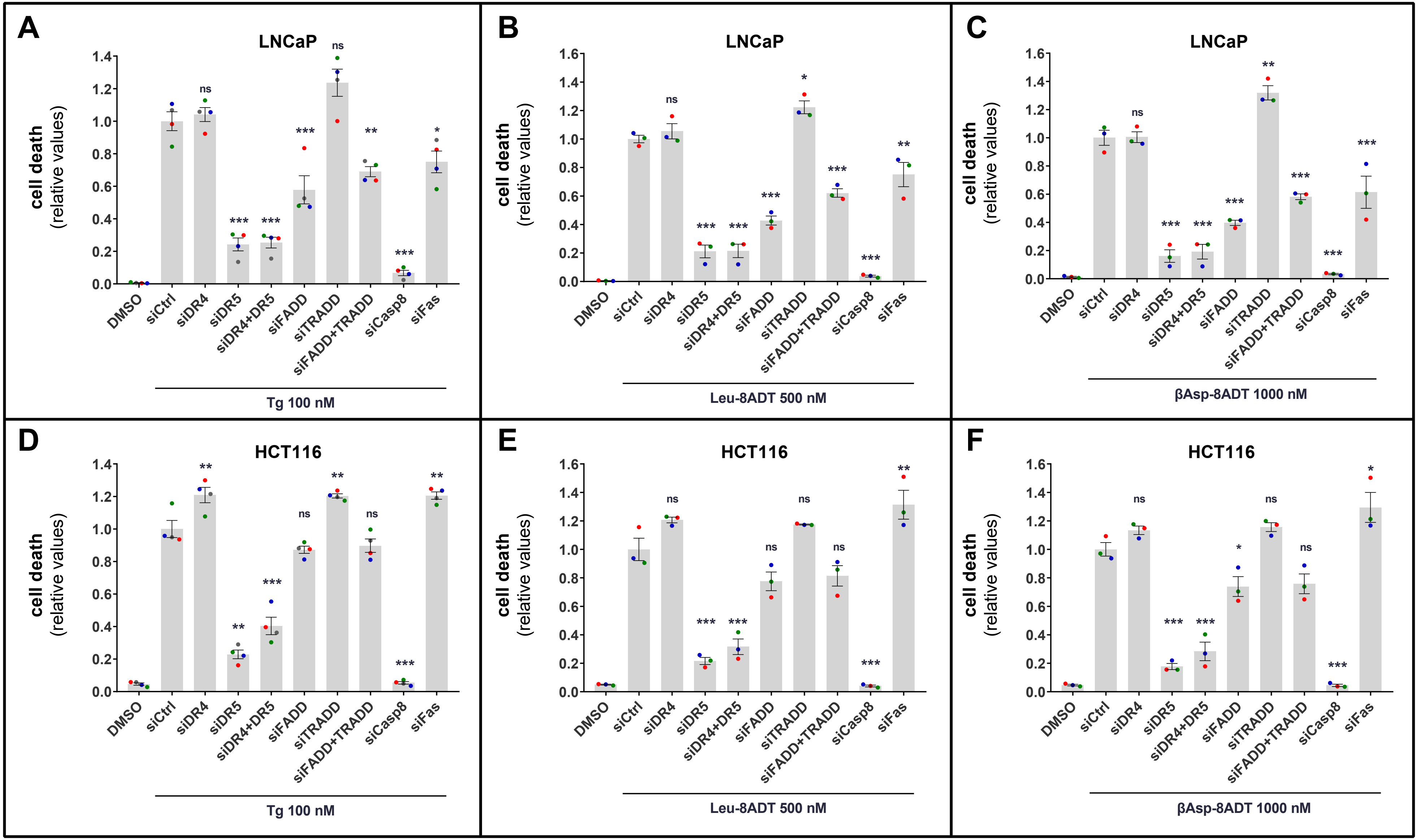

A
LNCaP
cell death (relative values)
DMSO siCtrl siDR4 siDR5 siDR4+DR5 siFADD siTRADD siFADD+TRADD siCasp8 siFas
Tg 100 nM
B
LNCaP
Leu-8ADT 500 nM
C
LNCaP
βAsp-8ADT 1000 nM
D
HCT116
Tg 100 nM
E
HCT116
Leu-8ADT 500 nM
F
HCT116
βAsp-8ADT 1000 nM

Figure S17

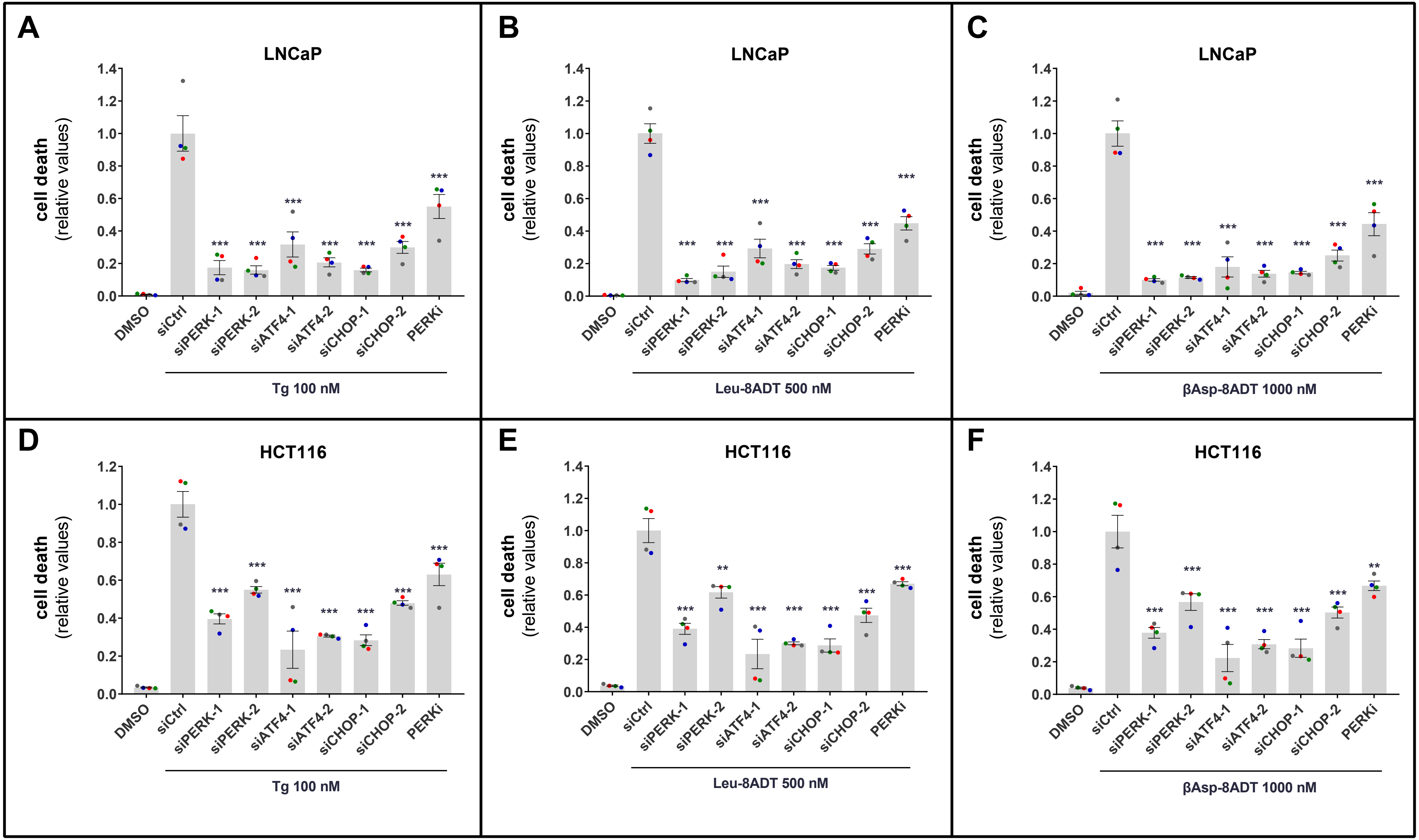

A
LNCaP
cell death
(relative values)
DMSO
siCtrl
siPERK-1
siPERK-2
siATF4-1
siATF4-2
siCHOP-1
siCHOP-2
PERKi
Tg 100 nM
B
LNCaP
Leu-8ADT 500 nM
C
LNCaP
βAsp-8ADT 1000 nM
D
HCT116
Tg 100 nM
E
HCT116
Leu-8ADT 500 nM
F
HCT116
βAsp-8ADT 1000 nM

Figure S18

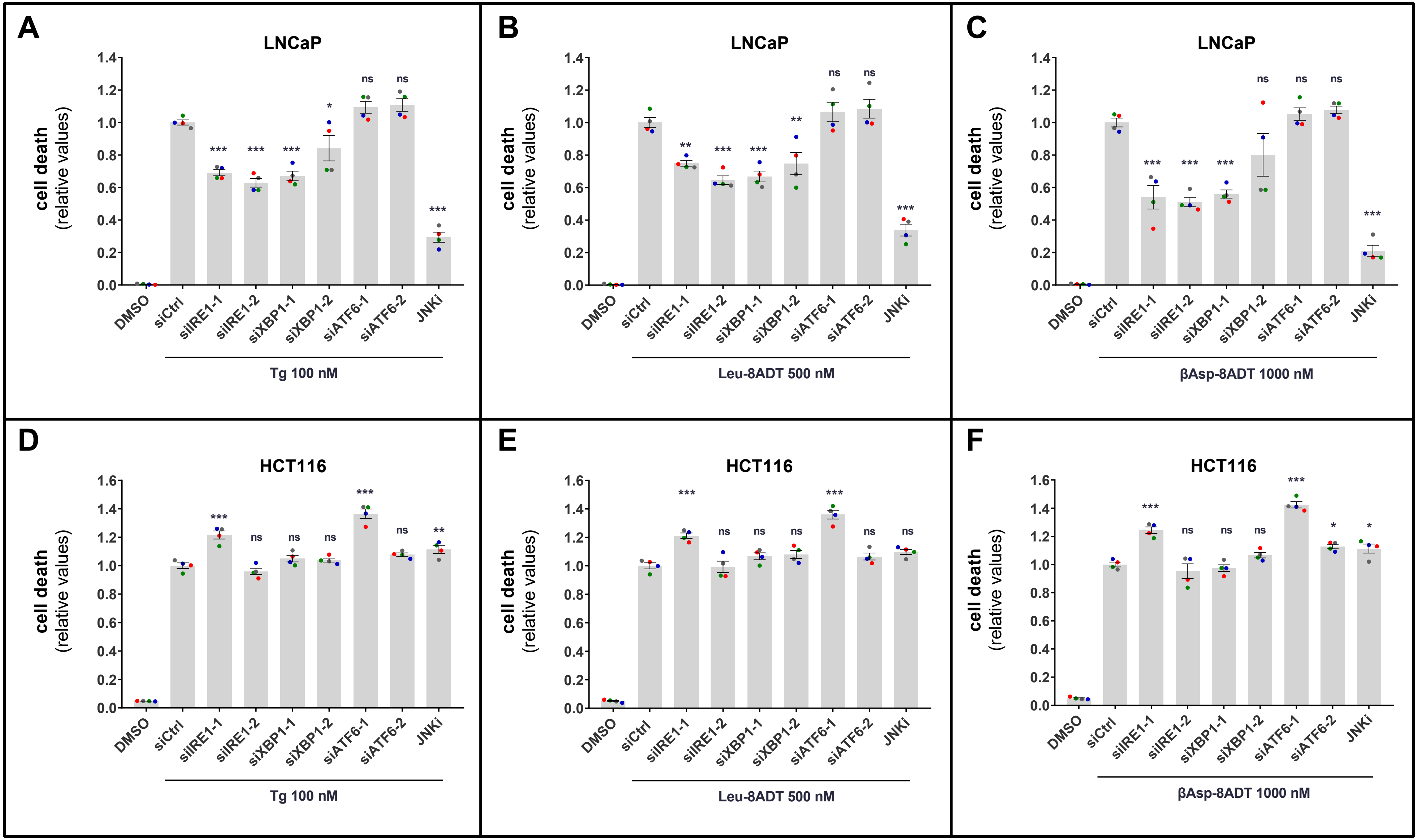

A
LNCaP
cell death
(relative values)
1.4
1.2
1.0
0.8
0.6
0.4
0.2
0.0
DMSO
siCtrl
siIRE1-1
siIRE1-2
siXBP1-1
siXBP1-2
siATF6-1
siATF6-2
JNKi
*** *** *** * ns ns ***
Tg 100 nM
B
LNCaP
** *** *** ** ns ns ***
Leu-8ADT 500 nM
C
LNCaP
*** *** *** ns ns ns ***
βAsp-8ADT 1000 nM
D
HCT116
1.6
*** ns ns ns *** ns **
Tg 100 nM
E
HCT116
*** ns ns ns *** ns ns
Leu-8ADT 500 nM
F
HCT116
*** ns ns ns *** * *
βAsp-8ADT 1000 nM
